# A Wearable Technology Delivering a Web-Based Diabetes Prevention Program to People at High Risk of Type 2 Diabetes: Randomized Controlled Trial

**DOI:** 10.2196/15448

**Published:** 2020-07-15

**Authors:** Emily Staite, Adam Bayley, Ebaa Al-Ozairi, Kurtis Stewart, David Hopkins, Jennifer Rundle, Neel Basudev, Zahra Mohamedali, Khalida Ismail

**Affiliations:** 1 Institute of Psychiatry, Psychology and Neurosciences King's College London London United Kingdom; 2 Faculty of Medicine Department of Medicine & Dasman Diabetes Institute Kuwait University Al Kuwayt Kuwait; 3 King's College Hospital NHS Foundation Trust King's Health Partners London United Kingdom; 4 South Thames Cleft Service St Thomas’ Hospital London United Kingdom; 5 Health Innovation Network London United Kingdom

**Keywords:** motivational interviewing, lifestyle, diabetes prevention program, theory of planned behavior, type 2 diabetes mellitus, wearable technology, mobile phone

## Abstract

**Background:**

Intensive lifestyle interventions are effective in reducing the risk of type 2 diabetes, but the implementation of learnings from landmark studies is expensive and time consuming. The availability of digital lifestyle interventions is increasing, but evidence of their effectiveness is limited.

**Objective:**

This randomized controlled trial (RCT) aimed to test the feasibility of a web-based diabetes prevention program (DPP) with step-dependent feedback messages versus a standard web-based DPP in people with prediabetes.

**Methods:**

We employed a two-arm, parallel, single-blind RCT for people at high risk of developing diabetes. Patients with a hemoglobin A_1c_ (HbA_1c_) level of 39-47 mmol/mol were recruited from 21 general practices in London. The intervention integrated a smartphone app delivering a web-based DPP course with SMS texts incorporating motivational interviewing techniques and step-dependent feedback messages delivered via a wearable device over 12 months. The control group received the wearable technology and access to the web-based DDP but not the SMS texts. As this was a feasibility study, the primary aim was to estimate potential sample size at different stages of the study, including the size of the target study population and the proportion of participants who consented, were randomized, and completed follow-up. We also measured the main outcomes for a full-scale RCT, namely, change in weight and physical activity at 6- and 12-month follow-ups, and secondary outcomes, including changes in the HbA_1c_ level, blood pressure, waist circumference, waist-to-hip ratio, and lipid levels.

**Results:**

We enrolled 200 participants: 98 were randomized to the intervention and 102 were randomized to the control group. The follow-up rate was higher in the control group (87/102, 85.3%) than in the intervention group (69/98, 70%) at 12 months. There was no treatment effect on weight at 6 months (mean difference 0.15; 95% CI −0.93 to 1.23) or 12 months (mean difference 0.07 kg; 95% CI −1.29 to 1.44) or for physical activity levels at 6 months (mean difference −382.90 steps; 95% CI −860.65 to 94.85) or 12 months (mean difference 92.64 steps; 95% CI −380.92 to 566.20). We did not observe a treatment effect on the secondary outcomes measured at the 6-month or 12-month follow-up. For the intervention group, the mean weight was 92.33 (SD 15.67) kg at baseline, 91.34 (SD 16.04) kg at 6 months, and 89.41 (SD 14.93) kg at 12 months. For the control group, the mean weight was 92.59 (SD 17.43) kg at baseline, 91.71 (SD 16.48) kg at 6 months, and 91.10 (SD 15.82) kg at 12 months. In the intervention group, the mean physical activity was 7308.40 (SD 4911.93) steps at baseline, 5008.76 (SD 2733.22) steps at 6 months, and 4814.66 (SD 3419.65) steps at 12 months. In the control group, the mean physical activity was 7599.28 (SD 3881.04) steps at baseline, 6148.83 (SD 3433.77) steps at 6 months, and 5006.30 (SD 3681.1) steps at 12 months.

**Conclusions:**

This study demonstrates that it is feasible to successfully recruit and retain patients in an RCT of a web-based DPP.

**Trial Registration:**

ClinicalTrials.gov NCT02919397; http://clinicaltrials.gov/ct2/show/NCT02919397

## Introduction

### Background

The prevalence of prediabetes is approximately 10% in the UK population [1], with higher rates in adults aged 40 years or older and those of South Asian or African Caribbean ethnicities [2]. Considering the rising prevalence of type 2 diabetes, which accounts for disproportionate and increasing costs to the individual, society, and health care systems globally [3,4], primary prevention is a current public health priority.

Landmark randomized controlled trials (RCTs) have repeatedly shown that intensive face-to-face diabetes prevention programs (DPP) are effective in reducing the risk of type 2 diabetes by approximately 50% [5-7]. The implementation of these landmark studies is expensive, time consuming for the patients and for health systems, and the uptake is often by those with the lowest risk for type 2 diabetes [8]. One solution is adapting the traditional DPP into a web-based DPP using wearable technologies and web-based programs. These are increasingly available from commercial providers at a low cost to the patient or health commissioners, but the evidence of their effectiveness in reducing the risk of type 2 diabetes is limited [9,10]. For example, in a recent RCT of 2062 people in India and the United Kingdom with impaired glucose tolerance, those who received 2 to 3 weekly SMS texts providing lifestyle advice did not have a significant reduction in diabetes conversion (defined by international criteria for fasting plasma glucose or hemoglobin [HbA_1c_] levels) compared with controls who received standard lifestyle advice at baseline only [11]. Similarly, a pilot pre-post noncontrolled study of a 16-week web-based DPP was associated with reduced weight and lower HbA_1c_ levels, improvements that persisted at the 2-year follow-up [12]. An RCT of a fully automated and algorithm-driven email, web and mobile DPP found significant improvements in biomedical outcomes, weight, and diabetes risk at 6-months compared with the wait list control group [13].

Components of behavior change techniques considered to be most effective in improving diet and physical activity (PA) are based on self-regulatory behaviors, such as goal setting, self-monitoring, giving feedback, utilizing social support, and motivational interviewing (MI) [14,15]. Interventions based on a psychological theory, for example, the theory of planned behavior, are considered to be more effective and have better outcomes in high-risk populations [16], although the minimum threshold of intensity, such as the number of sessions or messages or duration of the intervention, is not known [14,17,18].

### Aims

This RCT aimed to test the feasibility of a web-based DPP, consisting of a wearable technology that records PA, integrated with SMS texts based on MI techniques, and lifestyle education delivered via a smartphone app, over 12 months in participants with prediabetes. The primary aims were to assess (1) the potential size of the study population; (2) the proportion of those who consented to be screened for eligibility; (3) the proportion of those who were screened and who were eligible, consented, and randomized; (4) the proportion of those who were randomized and who completed the intervention; and (5) the proportion of those who completed the 6-month and 12-month follow-ups.

Our secondary aims were to measure the change in biomedical outcomes, including reducing weight and increasing PA, to inform the possible range of effect sizes and obtain outcome variance estimates required for sample size calculations in a full-scale trial.

## Methods

### Study Design

This was a two-arm, parallel, single-blind RCT conducted over 12 months. The trial has been reviewed and given favorable opinion by the London City and East Research Ethics Committee (16/LO/1505).

### Setting

We recruited patients from 3 clinical commissioning groups in London (Lambeth, Southwark, and Lewisham), which comprise a population of 912,687 residents, with one of the highest prevalence rates of type 2 diabetes [19] in England and with broad socioeconomic and ethnic diversity [20]. Patients were recruited from participating primary care surgeries with list sizes greater than 6000 patients.

### Participants

Patients with an HbA_1c_ level of 39 to 47 mmol/mol were defined as being in a prediabetes state according to the current American Diabetes Association criteria [21]. They were identified using a 2-stage process. First, patients at high risk of developing type 2 diabetes were identified by the general practitioner (GP) who conducted a search on the Egton Medical Information Systems (EMIS) web (clinical software where GPs can access patient health records) using HbA_1c_ results recorded in the previous 12 months. Additional data extracted from EMIS included anonymous ID, gender, date of birth, postcode, ethnic origin, QDiabetes score [22], and BMI. Potentially eligible patients (HbA_1c_ level of 39 to 47 mmol/mol) were invited to undergo screening for eligibility. All GPs used the same postal invitation. Second, the inclusion criteria at screening were as follows: adults aged between 18 and 65 years; BMI≥25 kg/m² (≥23 kg/m² if of Asian ethnicity) [19]; permanent residents in Lambeth, Southwark, or Lewisham; owning a smartphone (iPhone or Android models only)—defined as logging on at least once per day to the internet; being fluent in conversational English; and being ambulatory (eg, capable of walking without mobility support equipment).

The exclusion criteria included diabetes (not including past history of gestational diabetes); pregnancy; planning a pregnancy or lactating during the duration of the study; severe mental illness (severe depression with suicidal ideation, psychosis, bipolar affective disorder, dementia, learning difficulties, substance problem use, or dependence); severe physical disability (eg, that would prevent any increased uptake of physical exercise); advanced active disease, such as cancer or heart failure; any other condition that requires glucose-altering drugs; BMI≥50 kg/m²; and current participation in a weight loss program or DPP. When in doubt, we sought GP confirmation of eligibility.

### Baseline Measures

We collected sociodemographic data, including age, gender, postcode of residence, employment status, educational level, and self-reported ethnicity. On the basis of the participant’s postcode, we determined their indices of multiple deprivation (IMD) 2015 rank, which indicates the relative level of socioeconomic deprivation in their area [23].

#### Objective Physical Activity

Objective PA was measured using wearable technology (a wristband manufactured and provided by Buddi Ltd; *Wearable Technology* section below gives more details). Physical activity (number of steps per day) was recorded continuously by the wristband. Baseline PA was the mean step count of the first 7 days of wearing the wristband (starting from the baseline appointment). Days with no recorded steps were removed before calculating each participant’s mean step count (Multimedia Appendix 1 shows the number of days used in calculating step counts).

#### Biomedical Data

We collected HbA_1c_ (mmol/mol) and lipid levels (total cholesterol, high- and low-density lipoproteins, and fasting triglycerides; all values in mmol/L). Weight was measured in light clothing, without shoes, to 0.01 kg, and height to 0.1 cm using a stadiometer (Class 3 Tanita SC240). Weight and height measurements were used to calculate the BMI (kg/m^2^). Waist circumference (cm) was measured horizontally halfway between the lowest rib and the upper prominence of the pelvis using a nonextensible steel tape against the bare abdomen. Hip circumference was also measured to calculate the waist-to-hip ratio. Diastolic and systolic blood pressure (BP) and resting heart rate were measured with digital Omron BP monitors (Omron M7) using standardized procedures of the average of 2 readings taken 1 min apart while seated.

#### Self-Reported Data

Subjective PA was assessed using the International Physical Activity Questionnaire (IPAQ) [24], from which we derived 2 continuous summary scores (sitting minutes and total activity, given in metabolic equivalent of tasks min/week) and a categorical score (low, moderate, or high activity levels) of participants’ PA levels in the past week. Depressive symptoms were collected using the patient health questionnaire-9 (PHQ-9) [25]. Readiness to change was measured using the University of Rhode Island change assessment scale (URICA) [26], adapted from 32 items to 12 items for this study, to ask specifically about participants’ readiness to change dietary and activity behaviors with regard to their health; scores range from 2 to 14, with scores <8 indicating participants are in the *precontemplation* stage, 8 to 11 the *contemplation* stage, and >11 the *preparation or action* stage. Self-efficacy was measured using the self-efficacy for exercise scale, which has 9 items and a score range of 0 to 90, with higher scores indicating greater self-efficacy [27]. We collected data on smoking status and, if current, how many cigarettes per day. Alcohol intake was measured using the alcohol use disorders identification test (AUDIT) [28].

### Intervention

The intervention was based on the theory of planned behavior, which states that to change behavior, people need to form an intention [16]. Intention formation is influenced by 3 constructs: expected value or positive attitude (people see the value in making the change), subjective norm (significant others and peers also value the change), and self-efficacy (people believe they are capable of making the change).

#### Wearable Technology

All participants were issued with a wristband (manufactured and provided by Buddi Ltd), its charger, and instructions for operating the wristband and downloading the associated study-specific smartphone app. In the baseline appointment, participants downloaded the app onto their smartphone and wirelessly connected it to the wristband via Bluetooth with the help of the researcher if needed. Participants were told that they must maintain the Bluetooth connection to facilitate the transfer of data captured by the Buddi wristband to the participants’ smartphones. This allowed participants to track their activity in close to real time via the smartphone d as well as review past activity. If any technical issues arose, participants were able to contact a researcher (who was not blinded to the intervention allocation) for technical support, and any faulty devices were replaced.

#### Web-Based Education

We scheduled and delivered 22 web-based sessions over 12 months targeting diet, PA, and mental resilience. The curriculum was based on the newly developed Centers for Disease Control and Prevention PreventT2 curriculum and handouts [28], which is an implementation version of the original landmark DPP studies [5,29,30]. The web-based sessions were available through the smartphone app, with PDF transcripts available to download for each session. SMS texts were sent to participants via the smartphone app to notify them when each module of the web-based DPP was available (1 text every 1-4 weeks approximately).

#### Motivational SMS Texts

SMS texts were generated and delivered via the smartphone app using principles and techniques from MI to support participants in forming healthy intentions, encourage self-monitoring of lifestyle behaviors, and promote social support [15]. MI is normally a face-to-face collaborative conversation style for strengthening a person’s motivation, belief, and commitment to change. We adapted this principle for a virtual setting. The contents of the automated messages were temporally coordinated with the contents of the educational program. Participants typically received 3 to 4 MI-based messages/day (excluding Saturday and Sunday) for 12 months. There were 3 types of messages:

1. Messages targeting lifestyle behaviors encouraged *how* to make lifestyle changes (self-efficacy), for example, *Think about how many staircases you might be able to use today instead of the lift*, followed by *why* messages (expected value of change to the patient) as reinforcement, for example, *Exercise is best done little and often and will have a positive impact on your health*. The content was coordinated to mirror that of the educational program. One of each *how* and *why* message was sent daily.
2. One daily message giving feedback on the activity data received from the wearable technology; the content was based on the level of activity designed to reinforce or encourage an increase in activity levels.
3. Responsive messages were only sent if participants proactively selected the following on the app: *achieve* when they felt they had reached a goal, *crave* when they were thinking about pursuing an unhealthy behavior (eg, eating a high-calorie food or avoiding their exercise regime) but had not acted upon it, or *lapse* when they had acted upon their cravings and need support to re-engage with their good intentions. Participants also had the opportunity to record their achievements within the app.

#### Control Arm

The control group was provided with the Buddi wristband for the duration of the study and could access their activity data and web-based education material via the smartphone app. They received an automated message (via the smartphone app) informing them when the next educational session was available (ie, 22 messages in total), but they did not receive any other messages. This was weekly for modules 1 to 6, biweekly for modules 7 to 16, and monthly (4 weeks) for modules 17 to 22.

### Outcome Measures

For feasibility parameters, the primary outcomes were proportion recruited and randomized and proportion followed up. The primary clinical outcomes were change in weight (kg) and PA (mean steps per day) from baseline to 12 months, with an interim measure at 6 months. Follow-up PA was the mean step count of the 7 days of wear leading up to and including the day of the follow-up appointment.

The secondary outcomes were a change in HbA_1c_ levels and BP at 6 and 12 months and waist circumference, waist-to-hip ratio, and lipid levels at 12 months. The HbA_1c_ level was analyzed as a continuous and categorical variable, with the following categories: normal (<42 mmol/mol), prediabetes (39-47 mmol/mol), and diabetes (>47 mmol/mol).

### Sample Size

We aimed for 100 participants per arm, as this was a large enough sample to inform the practicalities of delivering the intervention, recruitment, uptake, and attrition to inform a full-scale trial rather than measures of intervention effects.

### Randomization and Allocation Concealment

Before randomization, participants wore the Buddi wristband for 1 week to familiarize with the technology and to collect baseline activity data. All patients were offered a brief educational session on the use of the Buddi device, and an instruction manual was provided. Randomization of participants was conducted by the data manager from an independent clinical trials unit using computer-generated randomization blocks of random sizes and stratified by surgery in a 1:1 ratio. Allocation concealment of the randomization list was held in a password-locked computer. This was an open-label study, but outcome assessors, laboratory technicians, and researchers entering and scoring the data were blinded to patients’ allocations.

### Statistical Analysis Plan

The full statistical analysis plan for this study is provided in Multimedia Appendix 2. Analysis and reporting were in line with the consolidated standards of reporting trials guidelines [29], including its extensions for pilot and feasibility trials; primary analyses were conducted on an intention-to-treat basis and using a two-sided significance level of .05. Statistical analyses were mainly descriptive, aiming to provide estimates of key feasibility parameters and to inform power calculations for a future definitive trial. Descriptive subanalyses were used to explore participation rates among participants based on ethnicity, education level, IMD 2015 score, BMI, depressive symptoms, readiness to change, and self-efficacy. The proportion of missing data for individual items and measures was examined to determine the suitability of instruments and the level of burden for a future full-scale trial. We compared baseline characteristics of (1) those who were eligible and who did and did not consent and (2) those who did and did not provide 12-month follow-up data for the primary clinical outcomes. The differences in treatment effect for the primary and secondary outcomes between the arms at 6- and 12-month follow-ups were analyzed using analysis of covariance–based, linear mixed effects models with prerandomization values as a covariate [30]; STATA’s *mixed* command was used for estimation.

## Results

### Trial Flow

**Figure 1:**
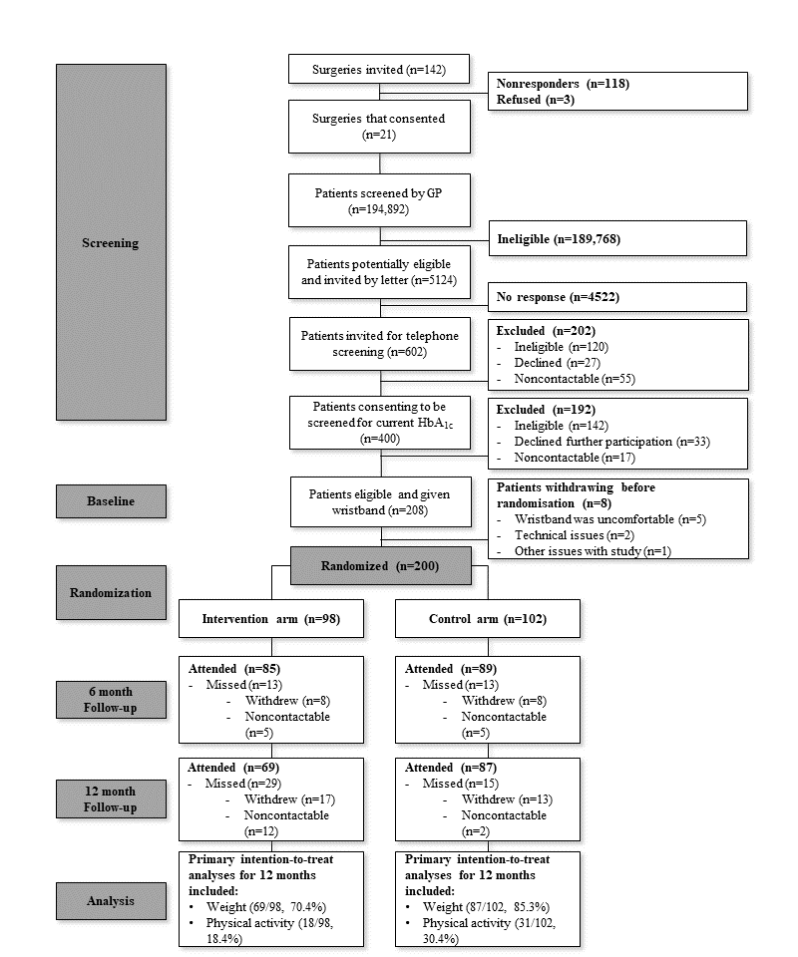
Participant flow diagram.

Figure 1 presents participant flow through the trial. Over a 6-month period, we sent postal invitations to 142 surgeries, whose patient list sizes were over 6000. A total 21 of 142 surgeries (14.8%) agreed to participate, from which 194,892 patients had the first-stage screening performed electronically by their GP. Out of these, 5124 were potentially eligible and invited for further screening by their GP by letter and telephone. The number of people who attended and consented for second-stage screening was approximately 11 patients per week, requiring 28 hours per week of a full-time equivalent research worker. Approximately half of those who attended the screening were eligible and consented to be randomized.

### Baseline Characteristics

The baseline characteristics of the participants are presented in Table 1. The two arms were broadly similar (except for ethnicity) and representative of the local catchment in terms of high levels of deprivation, with over half being of nonwhite ethnicity.

### Adherence to Intervention

Data were available for 192 participants (92 in the intervention arm). Of these 192, a total of 80 (80.0%) and 60 (65.2%) participants were adherent to the intervention in the intervention and control arms, respectively (X^2^_1_=4.6; *P*=.03). Participants used the smartphone app on average 41.5% (SD 27.5) and 33.2% (SD 27.5) of days in the control and intervention arms, respectively.

**Table 1:** Baseline characteristics.

Baseline characteristics		Intervention (n=98)^a^		Control (n=102)^b^		Total (N=200)	
Age (years), mean (SD)		51.76 (7.68)		52.78 (8.20)		52.28 (7.94)	
**Gender, n (%)**							
	Female		56 (57)		51 (50.0)		106 (53.0)
**Ethnicity, n (%)**							
	White		28 (28)		35 (34.3)		63 (31.5)
	African or African Caribbean		50 (51)		58 (56.9)		108 (54.0)
	Asian		16 (16)		4 (3.9)		20 (10.0)
	Other		4 (4)		5 (4.9)		9 (4.5)
**Highest qualification, n (%)**							
	No formal qualifications		3 (3)		5 (5.2)		8 (4.1)
	GCSE^c^ or equivalent		29 (29)		23 (23.7)		52 (26.8)
	A level or higher		65 (67)		69 (71.1)		134 (69.1)
Currently employed, full- or part-time, n (%)		74 (77)		78 (78.8)		152 (77.9)	
**Relationship status, n (%)**							
	Married/cohabiting		61 (62)		59 (57.8)		120 (60.0)
	Separated/divorced/widowed		12 (12)		13 (12.7)		25 (12.5)
	Single		25 (25)		30 (29.4)		55 (27.5)
**IMD^d^ 2015 quintiles, n (%)**							
	1—most deprived		33 (33)		36 (35.3)		69 (34.5)
	2		32 (32)		38 (37.3)		70 (35.0)
	3		21 (21)		19 (18.6)		40 (20.0)
	4		11 (11)		8 (7.8)		19 (9.5)
	5—least deprived		1 (1)		1 (1.0)		2 (1.0)
Family history of diabetes, n (%)		52 (53)		51 (50.0)		103 (51.5)	
**Smoking status, n (%)**							
	Current smoker		13 (13)		10 (9.8)		23 (11.5)
	Ex-smoker		32 (32)		41 (40.2)		73 (36.5)
	Nonsmoker		53 (54)		51 (50.0)		104 (52.0)
Number of cigarettes per day for current smokers, mean (SD)		7.50 (5.76)		6.40 (5.21)		7.02 (5.43)	
PHQ-9^e^ depression score, mean (SD)		4.04 (4.61)		4.25 (4.01)		4.15 (4.31)	
**AUDIT^f^ score category, n (%)**							
	Abstainer (0)		22 (22)		21 (20.6)		43 (21.5)
	Low risk (1-7)		71 (72)		69 (67.6)		140 (70.0)
	Possibly harmful (≥8)		5 (5)		12 (11.8)		17 (8.5)
IPAQ^g^ total physical activity, MET^h^ minutes/week, median (IQR)		2264.18 (2311.16)		2647.01 (2715.67)		2459.43 (2526.55)	
**URICA^i^ readiness to change, n (%)**							
	Precontemplation (<8)		22 (22.)		12 (11.8)		34 (17.1)
	Contemplation (8-11)		54 (55)		70 (68.6)		124 (62.3)
	Preparation or action (>11)		21 (21)		20 (19.6)		41 (20.6)
SEE^j^ self-efficacy score, mean (SD)		55.15 (22.11)		54.33 (21.95)		54.73 (21.97)	

^a^Number of missing cases for the intervention arm are as follows: highest qualification (n=1), currently employed (n=3), PHQ-9 (n=1), URICA (n=1), and SEE (n=2).

^b^Number of missing cases for the control arm are as follows: highest qualification (n=5) and currently employed (n=2).

^c^GCSE: general certificate of secondary education.

^d^IMD: indices of multiple deprivation.

^e^PHQ-9: patient health questionnaire-9.

^f^AUDIT: alcohol use disorders identification test.

^g^IPAQ: international physical activity questionnaire.

^h^MET: metabolic equivalent of the task.

^i^URICA: University of Rhode Island change assessment scale.

^j^SEE: self-efficacy for exercise.

### Summary of Outcome Data

Table 2 presents a descriptive summary of the clinical outcomes at each time point. We observed that the distributions for each primary outcome were positively skewed; however, log-transforming the data did not improve the distribution or alter the results of the analyses below. The pooled SD at 12 months for weight was 15.43 kg and for PA was 3588.76 steps.

When categorizing participants’ metabolic status based on their HbA_1c_ values, 3 (3.5%) and 5 (7.3%) in intervention group and 2 (2.2%) and 6 (6.9%) in the control group met the cut-off for diabetes (>47 mmol/mol) at 6 and 12 months, respectively. Participants with HbA_1c_ >47 mmol/mol were referred to the GP. Participants were informed of their results via telephone and told to contact their GP. A total of 4 (4.7%) and 9 (10.1%) participants returned to the normal range (<39 mmol/mol) in the intervention and control arms, respectively, at 6 months. At 12 months, 0 and 4 (4.6%) participants were in the normal range in the intervention and control arms, respectively. There was no difference in the proportion of participants who developed type 2 diabetes or who returned to normal HbA_1c_ levels between the 2 groups at 6 months (X^2^_2_=2.0; *P*=.36) or 12 months (X^2^_2_=3.2; *P*=.20).

**Table 2:** Summary of primary and secondary outcomes and pairwise comparisons.

Outcomes and time		Intervention arm			Control arm			Mean difference (95% CI)
		n	Mean (SD)	n		Mean (SD)		
**Weight (kg)**								
	Baseline	98	92.33 (15.67)	102		92.59 (17.43)	—^a^	
	6-month follow-up	85	91.34 (16.04)	89		91.71 (16.48)	0.15 (−0.93 to 1.23)^b^	
	12-month follow-up	69	89.41 (14.93)	87		91.10 (15.82)	0.07 (−1.29 to 1.44)	
**PA^c^ (mean steps per day)^d^**								
	Baseline	87	7308.40 (4911.93)	93		7599.28 (3881.04)	—	
	6-month follow-up	36	5008.76 (2733.22)	51		6148.83 (3433.77)	−382.90 (−860.65 to 94.85)	
	12-month follow-up	18	4814.66 (3419.65)	31		5006.30 (3681.1)	92.64 (−380.92 to 566.20)	
**BP^e^ diastolic (mm Hg)**								
	Baseline	98	82.92 (10.68)	102		81.47 (8.96)	—	
	6-month follow-up	82	81.41 (10.19)	89		83.22 (8.46)	−2.24 (−4.54 to 0.06)	
	12-month follow-up	68	83.03 (10.33)	87		83.87 (9.06)	−1.61 (−3.93 to 0.70)	
**BP systolic (mm Hg)**								
	Baseline	98	125.51 (17.39)	102		124.52 (12.19)	—	
	6-month follow-up	82	124.15 (16.33)	89		127.33 (13.30)	3.50 (−7.05 to 0.05)	
	12-month follow-up	68	125.37 (16.07)	87		127.54 (14.16)	−2.62 (−6.37 to 1.12)	
**Cholesterol:HDL^f^ ratio**								
	Baseline	97	4.02 (0.99)	102		4.14 (1.02)	—	
	12-month follow-up	68	4.06 (1.20)	86		3.97 (1.04)	0.11 (−0.08 to 0.30)	
**HbA_1c_ (mmol/mol)**								
	Baseline	98	42.27 (2.32)	102		42.29 (1.98)	—	
	6-month follow-up	85	42.12 (2.44)	89		41.82 (3.05)	0.20 (−0.50 to 0.91)	
	12-month follow-up	68	44.06 (2.31)	87		43.54 (2.68)	0.53 (−0.19 to 1.25)	
**HDL (mmol/L)**								
	Baseline	97	1.39 (0.33)	102		1.34 (0.33)	—	
	12-month follow-up	68	1.39 (0.32)	86		1.38 (0.31)	0.00 (−0.07 to 0.06)	
**LDL^g^ (mmol/L)**								
	Baseline	96	3.33 (0.84)	101		3.28 (0.83)	—	
	12-month follow-up	67	3.30 (0.85)	85		3.28 (0.85)	0.02 (−0.18 to 0.23)	
**Total cholesterol (mmol/L)**								
	Baseline	97	5.35 (0.96)	102		5.30 (0.93)	—	
	12-month follow-up	68	5.37 (1.03)	86		5.28 (0.99)	0.10 (−0.11 to 0.31)	
**Triglycerides (mmol/L)**								
	Baseline	97	1.39 (1.14)	102		1.39 (0.86)	—	
	12-month follow-up	68	1.72 (3.13)	86		1.35 (0.84)	0.39 (−0.02 to 0.81)	
**Waist circumference (cm)**								
	Baseline	97	103.31 (12.21)	100		103.92 (12.32)	—	
	12-month follow-up	69	96.73 (11.69)	86		97.38 (10.99)	−0.60 (−2.45 to 1.26)	
**Waist-to-hip ratio**								
	Baseline	97	0.92 (0.07)	100		0.93 (0.08)	—	
	12-month follow-up	69	0.91 (0.08)	86		0.90 (0.08)	0.00 (−0.02 to 0.02)	

^a^Baseline comparisons are not done, as per the statistical analysis plan noted above.

^b^Pairwise comparison outputs were calculated by subtracting the control arm from the intervention arm, so a negative value indicates the control arm had a higher mean.

^c^PA: physical activity.

^d^Number of days included in the step count calculations is given in Multimedia Appendix 1.

^e^BP: blood pressure.

^f^HDL: high-density lipoprotein.

^g^LDL: low-density lipoprotein.

### Primary Analyses

The fixed and random effects of the mixed effects models for weight and PA are presented in Multimedia Appendix 3. There was no treatment effect on weight or PA at 6 or 12 months (Table 2), or for HbA_1c_ levels, waist circumference, waist-to-hip ratio, lipid levels, or BP at 12 months.

### Sensitivity Analyses

Our sensitivity analyses adjusting for baseline characteristics did not alter our conclusions of the primary outcome analysis above. Readiness to change (URICA stage) did not moderate the treatment-by-time effect for weight (*F*_1,2_=0.10; *P*=.90) or PA (*F*_1,2_=0.56; *P*=.57).

Our analysis of responders to the control or intervention arm indicated that improvements in weight and/or PA were associated with baseline smoking status (X^2^_2_=11.6; *P*=.003) and PHQ-9 categorical score (X^2^_4_=10.7; *P*=.03; Multimedia Appendix 4).

Testing the associations between IPAQ scores and mean step counts revealed a significant positive correlation between the intervention arm’s IPAQ total activity score and step counts at baseline only (*r*_85_=0.22; *P*=.04; Multimedia Appendix 5). The series of 1-way analysis of variances showed that the IPAQ activity category (low, moderate, or high) was only associated with step counts for the intervention arm at baseline (*F*_2,84_=4.83; *P*=.01).

## Discussion

We have demonstrated that conducting an RCT that tested web-based DPP delivered via a wearable technology and a smartphone app is feasible in terms of participation and retention of study patients over a period of 12 months.

### Principal Findings

We successfully reached our target sample size in 36 weeks, with a recruitment rate of 5 to 6 patients per week per full-time research worker. For full-scale RCT testing the intervention with an expected effect size of 0.1 and 90% power, we estimate recruitment will take about 3.5 years per research worker or just over 1 year for 3 research workers (Multimedia Appendix 6).

We piloted the statistical plan that we would anticipate using for a full-scale RCT, which appeared to be valid, although hypothesis testing was only a secondary aim. The only significant difference observed was that those in the intervention group had lower PA levels at 6 months compared with those in the control group, but they were also less likely to complete the intervention and more likely to be noncontactable or have withdrawn at follow-up.

An important difference between our study and previous RCTs [11] is that we showed that it is feasible to use a wearable technology that continually records levels of PA (ie, step count). Furthermore, our study included male and female participants compared with another study that included only male participants [31]. We also had higher rates of retention than those in other studies. For instance, in an RCT of a fully automated and algorithm-driven web-based DPP [13], 72% of intervention participants were still interacting with the program at 6 months. We achieved 87% of participants attending their 6-month follow-up.

### Strengths and Limitations

A key strength is that this is the first feasibility study in the United Kingdom, which tests an automated web-based DPP designed to mirror the landmark face-to-face DPPs. The sample was representative of the ethnic and social diversity common in prediabetes. We noted that there was variation by GP surgery in response, and this suggests that clustering should be considered in any full-scale RCT.

An important observation is the importance of sustaining the functionality of the technology. Despite a prior *road-test* of the wearable technology and its connectivity to the smartphone app by the commercial provider in a handful of volunteers, there were mechanical and technical failures that may have resulted in participants randomized to the intervention arm receiving less of the required *dose* and withdrawing because of these issues. For example, we observed a sharp decrease in the mean step counts for participants at 6 months and 12 months compared with baseline, suggesting that the wristband may not have been accurately recording PA over time or that participants were unaware of ongoing technical issues. Other explanations could be that participants reduced their step count over time despite being in the intervention group or that participants reduced the amount of time they wore the device.

Participant adherence to the intervention could not be comprehensively documented as the technology for this did not exist; therefore, we were not able to capture how the participants used the app, particularly if they accessed the DPP education materials (and for how long) or if they read the SMS messages sent to them.

The follow-up rates for the primary outcomes were, on average, 75%, and predictors of missing data included higher BMI, presence of depressive symptoms, and current smokers (ie, those more at risk of developing type 2 diabetes and increasing the risk of underestimation).

We assessed the degree to which the wristband-derived step counts (ie, objectively measured PA) were associated with a validated self-reported measure of PA levels. We observed that the 2 measures only corresponded to a limited extent in the control arm, with step counts being weakly and positively correlated with total self-reported activity at 2 time points. The self-reported scores were not associated with step counts in the intervention arm. There are several possible reasons for this discrepancy, one being that these participants disengaged from the intervention (ie, wearing the wristband because of the technical problems or the higher intensity of messages).

The responses of the participants to our implementation processes questionnaire were generally favorable of the intervention; however, they did note several key areas for improvement for a full-scale RCT. These included improving the wristband’s clasp, offering the wristband in different styles, and improving the app’s accessibility (eg, offering the educational material in other digital formats) and formatting (eg, improving the readability and precision of the activity graphs and personalizing the content and frequency of messages).

### Research and Clinical Implications

This study found that there is a sufficiently large target population of patients for screening and a reasonably good participation rate (ie, patients are keen to receive support for diabetes prevention). Ensuring an optimum balance in the intensity of information sent and the functionality of the technology are potential key components to consider for a full-scale RCT.

## Appendix

Multimedia Appendix 1 Number of days used in calculating step counts.

Multimedia Appendix 2 Full statistical analysis plan.

Multimedia Appendix 3 Fixed and random effects of the mixed effects models for weight and PA.

Multimedia Appendix 4 Analysis of responders.

Multimedia Appendix 5 Testing associations between the International Physical Activity Questionnaire scores and mean step count.

Multimedia Appendix 6 Sample size calculations for a future full-scale randomized controlled trial.

Multimedia Appendix 7 CONSORT-eHEALTH (V 1.6.1).

## Abbreviations

AUDIT: alcohol use disorders identification test
BP: blood pressure
CDC: Centers for Disease Control and Prevention
DPP: diabetes prevention program
EMIS: Egton Medical Information Systems
GP: general practitioner
HbA_1c_: hemoglobin A_1c_
IMD: indices of multiple deprivation
IPAQ: International Physical Activity Questionnaire
KCL: King’s College London
MI: motivational interviewing
PHQ-9: patient health questionnaire-9
RCT: randomized controlled trial
URICA: University of the Rhode Island change assessment scale

## References

[1] Public Health England (2015). Government of UK Developer Documentation.

[2] Mainous A, Tanner R, Baker R, Zayas C, Harle CA (2014). Prevalence of prediabetes in England from 2003 to 2011: population-based, cross-sectional study. BMJ Open.

[3] Hex N, Bartlett C, Wright D, Taylor M, Varley D (2012). Estimating the current and future costs of type 1 and type 2 diabetes in the UK, including direct health costs and indirect societal and productivity costs. Diabet Med.

[4] Seuring T, Archangelidi O, Suhrcke M (2015). The economic costs of type 2 diabetes: a global systematic review. Pharmacoeconomics.

[5] Knowler WC, Barrett-Connor E, Fowler SE, Hamman RF, Lachin JM, Walker EA, Nathan DM, Diabetes Prevention Program Research Group (2002). Reduction in the incidence of type 2 diabetes with lifestyle intervention or metformin. N Engl J Med.

[6] Lindström J, Peltonen M, Eriksson J, Ilanne-Parikka P, Aunola S, Keinänen-Kiukaanniemi S, Uusitupa M, Tuomilehto J, Finnish Diabetes Prevention Study (DPS) (2013). Improved lifestyle and decreased diabetes risk over 13 years: long-term follow-up of the randomised Finnish diabetes prevention study (DPS). Diabetologia.

[7] Pan X, Li G, Hu Y, Wang J, Yang W, An Z, Hu Z, Lin J, Xiao J, Cao H, Liu P, Jiang X, Jiang Y, Wang J, Zheng H, Zhang H, Bennett PH, Howard BV (1997). Effects of diet and exercise in preventing NIDDM in people with impaired glucose tolerance. The Da Qing IGT and diabetes study. Diabetes Care.

[8] Aguiar E, Morgan P, Collins C, Plotnikoff R, Callister R (2014). Efficacy of interventions that include diet, aerobic and resistance training components for type 2 diabetes prevention: a systematic review with meta-analysis. Int J Behav Nutr Phys Act.

[9] Ma J, Yank V, Xiao L, Lavori P, Wilson S, Rosas L, Stafford RS (2013). Translating the diabetes prevention program lifestyle intervention for weight loss into primary care: a randomized trial. JAMA Intern Med.

[10] Johnson M, Jones R, Freeman C, Woods H, Gillett M, Goyder E, Payne N (2013). Can diabetes prevention programmes be translated effectively into real-world settings and still deliver improved outcomes? A synthesis of evidence. Diabet Med.

[11] Nanditha A, Thomson H, Susairaj P, Srivanichakorn W, Oliver N, Godsland IF, Majeed A, Darzi A, Satheesh K, Simon M, Raghavan A, Vinitha R, Snehalatha C, Westgate K, Brage S, Sharp SJ, Wareham NJ, Johnston DG, Ramachandran A (2020). A pragmatic and scalable strategy using mobile technology to promote sustained lifestyle changes to prevent type 2 diabetes in India and the UK: a randomised controlled trial. Diabetologia.

[12] Sepah SC, Jiang L, Peters AL (2015). Long-term outcomes of a web-based diabetes prevention program: 2-year results of a single-arm longitudinal study. J Med Internet Res.

[13] Block G, Azar K, Romanelli R, Block T, Hopkins D, Carpenter H, Dolginsky M, Hudes M, Palaniappan L, Block CH (2015). Diabetes prevention and weight loss with a fully automated behavioral intervention by email, web, and mobile phone: a randomized controlled trial among persons with prediabetes. J Med Internet Res.

[14] Baker M, Simpson K, Lloyd B, Bauman A, Singh MA (2011). Behavioral strategies in diabetes prevention programs: a systematic review of randomized controlled trials. Diabetes Res Clin Pract.

[15] Miller W, Rollnick S (2002). Motivational Interviewing: Preparing People for Change.

[16] Ajzen I (1991). The theory of planned behavior. Organ Behav Hum Decis Process.

[17] Ebrahim S, Taylor F, Ward K, Beswick A, Burke M, Smith DG (2011). Multiple risk factor interventions for primary prevention of coronary heart disease. Cochrane Database Syst Rev.

[18] Greaves CJ, Sheppard KE, Abraham C, Hardeman W, Roden M, Evans PH, Schwarz P, IMAGE Study Group (2011). Systematic review of reviews of intervention components associated with increased effectiveness in dietary and physical activity interventions. BMC Public Health.

[19] (2017). NHS Digital.

[20] Office of National Statistics.

[21] American Diabetes Association (2018). 2 Classification and diagnosis of diabetes. Diabetes Care.

[22] Hippisley-Cox J, Coupland C, Robson J, Sheikh A, Brindle P (2009). Predicting risk of type 2 diabetes in England and Wales: prospective derivation and validation of QDScore. Br Med J.

[23] (2015). Government of UK.

[24] Craig CL, Marshall AL, Sjöström M, Bauman AE, Booth ML, Ainsworth BE, Pratt M, Ekelund U, Yngve A, Sallis JF, Oja P (2003). International physical activity questionnaire: 12-country reliability and validity. Med Sci Sports Exerc.

[25] Spitzer R, Kroenke K, Williams JB (1999). Validation and utility of a self-report version of PRIME-MD: the PHQ primary care study. Primary care evaluation of mental disorders. Patient health questionnaire. J Am Med Assoc.

[26] Dozois D, Westra H, Collins K, Fung T, Garry Jk (2004). Stages of change in anxiety: psychometric properties of the University of Rhode Island change assessment (URICA) scale. Behav Res Ther.

[27] Resnick B, Jenkins LS (2000). Testing the reliability and validity of the Self-Efficacy for Exercise scale. Nurs Res.

[28] Saunders J, Aasland O, Babor T, de la Fuente JR, Grant M (1993). Development of the alcohol use disorders identification test (AUDIT): WHO collaborative project on early detection of persons with harmful alcohol consumption--II. Addiction.

[29] Campbell MK, Elbourne DR, Altman DG, CONSORT group (2004). CONSORT statement: extension to cluster randomised trials. Br Med J.

[30] Bauer DJ, Sterba SK, Hallfors DD (2008). Evaluating group-based interventions when control participants are ungrouped. Multivariate Behav Res.

[31] Ramachandran A, Snehalatha C, Ram J, Selvam S, Simon M, Nanditha A, Shetty AS, Godsland If, Chaturvedi N, Majeed A, Oliver N, Toumazou C, Alberti KG, Johnston DG (2013). Effectiveness of mobile phone messaging in prevention of type 2 diabetes by lifestyle modification in men in India: a prospective, parallel-group, randomised controlled trial. Lancet Diabetes Endocrinol.

