# Supplementary material for: A Wearable Technology Delivering a Web-Based Diabetes Prevention Program to People at High Risk of Type 2 Diabetes: Randomized Controlled Trial
Source: JMIR Mhealth Uhealth. 2020 Jul 15;8(7):e15448. doi: 10.2196/15448 (PMC7391669; doi:10.2196/15448)
Supplement: Multimedia Appendix 1 [file mhealth_v8i7e15448_app1.docx]

### Physical activity (step count) calculation

| Table S1. Days of recorded steps included in the mean step count calculations. | | |
| --- | --- | --- |
|  | Control | Intervention |
| Baseline | 6.18 (1.44), n=93 | 6.24 (1.28), n=87 |
| 6 months | 5.08 (2.54), n=51 | 4.53 (2.42), n=36 |
| 12 months | 5.32 (2.02), n=31 | 4.33 (2.35), n=18 |

| Table S2. Summary of physical activity (mean steps per day) for participants with seven days of recorded activity. | | |
| --- | --- | --- |
|  | Control | Intervention |
| Baseline | 7914.69 (3166.99), n=62 | 8267.98 (5567.48), n=56 |
| 6 months | 7413.73 (2707.27), n=28 | 5448.18 (1954.75), n=13 |
| 12 months | 6922.92 (3290.59), n=13 | 8452.07 (4858.81), n=4 |
