# Supplementary material for: A Wearable Technology Delivering a Web-Based Diabetes Prevention Program to People at High Risk of Type 2 Diabetes: Randomized Controlled Trial
Source: JMIR Mhealth Uhealth. 2020 Jul 15;8(7):e15448. doi: 10.2196/15448 (PMC7391669; doi:10.2196/15448)
Supplement: Multimedia Appendix 2 [file mhealth_v8i7e15448_app2.docx]

## Statistical analysis plan

Analysis and reporting were in line with the Consolidated Standards of Reporting Trials (CONSORT) guidelines [29], including its extensions for pilot and feasibility trials, with primary analyses being on an intention-to-treat basis and a 0.05 two-sided significance level. Statistical analyses are mainly descriptive, aiming to provide estimates of key feasibility parameters and to inform power calculations for a future definitive trial. A description of the sample is presented using means and standard deviations (SD) for continuous data and frequencies and proportions for categorical variables. Descriptive sub-analyses were used to explore participation rates among participants based on ethnicity, education level, IMD 2015 score, BMI, depressive symptoms, readiness to change, and self-efficacy. The amount of missing data for individual items and entire measures were examined to determine the suitability of instruments and the level of burden for a future full-scale trial. We compared baseline characteristics of: a) those who were eligible who did and did not consent; and b) those who did and did not provide 12-month follow-up data for the primary outcome.

Adherence to the intervention was calculated by determining the proportion of days that a participant used the smartphone application and had a wristband connected to it for at least one hour per day. We considered a threshold of ≥14% of days that the participant had their wristband and it was registered with Buddi’s system with at least one hour of usage, from baseline to the participant’s last follow-up or withdrawal, as being minimally adherent to the intervention.

The differences in treatment effect for the primary and secondary outcomes between the arms at 6-month and 12-month follow-ups were analyzed using ANCOVA-based, linear mixed-effects models with pre-randomisation values as a covariate [30]. STATA’s *mixed* command was used for the estimation.

The following sensitivity analysis was conducted to determine if any violations of the assumption of data missing at random (MAR) affected the outcomes. For any demographic or clinical baseline variables that were predictors of outcome missingness, then those variables were included as covariates in the primary analysis model and post-treatment group difference estimates and associated confidence intervals were examined for differences.

We conducted a *post hoc* comparison of participants’ baseline characteristics for those that showed any improvements in either of the two primary outcomes at the 12-month follow-up. We used Student’s t-tests and χ^2^-tests to compare continuous and categorical variables, respectively.
