# Supplementary material for: A Wearable Technology Delivering a Web-Based Diabetes Prevention Program to People at High Risk of Type 2 Diabetes: Randomized Controlled Trial
Source: JMIR Mhealth Uhealth. 2020 Jul 15;8(7):e15448. doi: 10.2196/15448 (PMC7391669; doi:10.2196/15448)
Supplement: Multimedia Appendix 3 [file mhealth_v8i7e15448_app3.docx]

| **Table S3. Fixed and random effects for the primary outcomes.** | | |
| --- | --- | --- |
| ***Fixed effects*** | **Weight (kg)** | **PA (mean steps/day)** |
| *Time-by-arm* |  |  |
| 6-month by Control | -0.15 (-1.23 to 0.93) | 382.90 (-94.85 to 860.65) |
| 12-month by Intervention | -0.60 (-1.35 to 0.16) | 116.86 (-304.21 to 537.94) |
| 12-month by Control | -0.67 (-1.88 to 0.54) | 24.23 (-438.69 to 487.15) |
| *Time-by-baseline* |  |  |
| 6-month | 0.96 (0.93 to 1.00) | 0.05 (-0.01 to 0.11) |
| 12-month | 0.92 (0.88 to 0.96) | 0.07 (0.01 to0.12) |
| Constant | 92.20 (91.43 to 92.98) | 6353.85(5906.99 to 6800.71) |
| ***Random effects*** |  |  |
| Constant variance (e1) | 13.26 (10.74 to 16.37) | 2486017 (2006711 to 3079806) |
| Constant variance (e2) | 19.91 (15.94 to 24.88) | 2220861 (1774268 to 2779864) |
| Residual covariance | 11.49 (8.45 to 14.54) | 687498.8 (312211.3 to 1062786) |
| *Cell data present coefficients (95% CI) for fixed effects or estimates (95% CI) for random effects.*  The weight model included 330 observations, 177 groups, and an average of 1.9 observations per group. The PA model included 325 observations, 174 groups, and an average of 1.9 observations per group. | | |
