# Supplementary material for: A Wearable Technology Delivering a Web-Based Diabetes Prevention Program to People at High Risk of Type 2 Diabetes: Randomized Controlled Trial
Source: JMIR Mhealth Uhealth. 2020 Jul 15;8(7):e15448. doi: 10.2196/15448 (PMC7391669; doi:10.2196/15448)
Supplement: Multimedia Appendix 4 [file mhealth_v8i7e15448_app4.docx]

### Summary of responder analysis

| Table S4. Summary of continuous baseline variables. | | | |
| --- | --- | --- | --- |
|  | Non-responder | Responder | p-value |
| Age | 51.31 (8.47), n=106 | 53.35 (7.21), n=94 | 0.067 |
| AUDIT score | 3.06 (3.31), n=106 | 2.89 (3.89), n=94 | 0.751 |
| BMI | 32.29 (4.88), n=106 | 33.18 (5.44), n=94 | 0.226 |
| Self-efficacy total score | 52.72 (22.65), n=105 | 56.99 (21.08), n=93 | 0.171 |
| HbA1c | 42.10 (2.15), n=106 | 42.48 (2.14), n=94 | 0.219 |
| HDL cholesterol | 1.34 (0.32), n=105 | 1.39 (0.35), n=94 | 0.286 |
| HDL:total cholesterol ratio | 4.12 (1.04), n=105 | 4.03 (0.97), n=94 | 0.546 |
| IMD score | 28.39 (5.52), n=106 | 28.86 (5.57), n=94 | 0.557 |
| IPAQ sitting minutes | 331.51 (226.61), n=106 | 350.74 (205.69), n=94 | 0.530 |
| IPAQ total activity score | 2310.77 (2436.34), n=106 | 2627.05 (2627.54), n=94 | 0.380 |
| LDL cholesterol | 3.28 (0.84), n=104 | 3.33 (0.83), n=93 | 0.687 |
| PHQ-9 score | 4.06 (4.14), n=105 | 4.30 (4.51), n=94 | 0.696 |
| Total cholesterol | 5.31 (0.95), n=105 | 5.34 (0.94), n=94 | 0.834 |
| URICA score | 9.67 (1.82), n=105 | 9.59 (2.11), n=94 | 0.781 |
| Waist circumference | 102.44 (11.63), n=104 | 104.94 (12.82), n=93 | 0.154 |
