# Supplementary material for: A Wearable Technology Delivering a Web-Based Diabetes Prevention Program to People at High Risk of Type 2 Diabetes: Randomized Controlled Trial
Source: JMIR Mhealth Uhealth. 2020 Jul 15;8(7):e15448. doi: 10.2196/15448 (PMC7391669; doi:10.2196/15448)
Supplement: Multimedia Appendix 5 [file mhealth_v8i7e15448_app5.docx]

| Table S5. Summary of categorical baseline variables. | | | | |
| --- | --- | --- | --- | --- |
|  |  | Non-responder | Responder | p-value |
| AUDIT category | Abstainer | 19 (17.9%) | 24 (25.5%) | 0.260 |
|  | Possibly harmful | 8 (7.5%) | 10 (10.6%) |  |
|  | Low risk | 79 (74.5%) | 60 (63.8%) |  |
| Education | 1 | 6 (5.8%) | 2 (2.2%) | 0.259 |
|  | 2 | 5 (4.8%) | 2 (2.2%) |  |
|  | 3 | 28 (27.2%) | 17 (18.5%) |  |
|  | 4 | 25 (24.3%) | 30 (32.6%) |  |
|  | 5 | 28 (27.2%) | 26 (28.3%) |  |
|  | 6 | 11 (10.7%) | 15 (16.3%) |  |
| Employment status | Unemployed | 20 (19.2%) | 25 (26.9%) | 0.268 |
|  | Part- or full-time employed | 84 (80.8%) | 68 (73.1%) |  |
| Ethnicity | Asian | 12 (11.3%) | 8 (8.5%) | 0.057 |
|  | Black | 65 (61.3%) | 43 (45.7%) |  |
|  | Other | 3 (2.8%) | 6 (6.4%) |  |
|  | White | 26 (24.5%) | 37 (39.4%) |  |
| Family history of diabetes | No | 55 (51.9%) | 43 (45.7%) | 0.468 |
|  | Yes | 51 (48.1%) | 51 (54.3%) |  |
| GP borough | 1 | 15 (14.2%) | 21 (22.3%) | 0.280 |
|  | 2 | 24 (22.6%) | 22 (23.4%) |  |
|  | 3 | 67 (63.2%) | 51 (54.3%) |  |
| IMD quintile | 1 | 36 (34.0%) | 33 (35.1%) | 0.412 |
|  | 2 | 43 (40.6%) | 27 (28.7%) |  |
|  | 3 | 18 (17.0%) | 22 (23.4%) |  |
|  | 4 | 8 (7.5%) | 11 (11.7%) |  |
|  | 5 | 1 (0.9%) | 1 (1.1%) |  |
| IPAQ category | High | 31 (29.2%) | 35 (37.2%) | 0.482 |
|  | Low | 22 (20.8%) | 18 (19.1%) |  |
|  | Moderate | 53 (50.0%) | 41 (43.6%) |  |
| PHQ-9 category | Mild | 21 (20.0%) | 26 (27.7%) | 0.031 |
|  | Moderate-severe | 1 (0.9%) | 3 (3.2%) |  |
|  | Moderate | 14 (13.3%) | 2 (2.1%) |  |
|  | None | 68 (64.8%) | 61 (64.9%) |  |
|  | Severe | 1 (0.9%) | 2 (2.1%) |  |
| Relationship status | Married or partnership | 65 (61.3%) | 55 (58.5%) | 0.910 |
|  | Separated/divorced/widowed | 13 (12.3%) | 13 (13.8%) |  |
|  | Single | 28 (26.4%) | 26 (27.7%) |  |
| Sex | Female | 51 (48.1%) | 56 (59.6%) | 0.139 |
|  | Male | 55 (51.9%) | 38 (40.4%) |  |
| Smoking status | Current | 19 (17.9%) | 4 (4.3%) | 0.003 |
|  | Ex-smoker | 41 (38.7%) | 32 (34.0%) |  |
|  | Never | 46 (43.4%) | 58 (61.7%) |  |
| URICA category | Pre-contemplation | 17 (16.2%) | 17 (18.1%) | 0.748 |
|  | Contemplation | 68 (64.8%) | 56 (59.6%) |  |
|  | Preparing or action | 20 (19.1%) | 21 (22.3%) |  |
| Treatment arm | Control | 47 (44.3%) | 55 (58.5%) | 0.063 |
|  | Intervention | 59 (55.7%) | 39 (41.5%) |  |

### Descriptive summary of IPAQ scores

| Table S6. Descriptive summary of IPAQ total physical activity and sitting scores by arm and time point. | | | | | | | |
| --- | --- | --- | --- | --- | --- | --- | --- |
|  |  | **Control** | | | **Intervention** | | |
| **IPAQ score** | **Time** | **Median** | **IQR** | N | **Median** | **IQR** | N |
| Sitting (minutes) | Baseline | 360 | 270.0 | 102 | 330 | 300.0 | 98 |
| Sitting (minutes) | 6 months | 300 | 210.0 | 89 | 360 | 300.0 | 85 |
| Sitting (minutes) | 12 months | 300 | 300.0 | 87 | 360 | 300.0 | 69 |
| Total (MET-minutes/week) | Baseline | 2066 | 2889.5 | 102 | 1453 | 2227.5 | 98 |
| Total (MET-minutes/week) | 6 months | 2373 | 3540.0 | 89 | 2026 | 3648.0 | 85 |
| Total (MET-minutes/week) | 12 months | 2496 | 2758.5 | 87 | 1782 | 3132.0 | 69 |
