# Supplementary material for: A Wearable Technology Delivering a Web-Based Diabetes Prevention Program to People at High Risk of Type 2 Diabetes: Randomized Controlled Trial
Source: JMIR Mhealth Uhealth. 2020 Jul 15;8(7):e15448. doi: 10.2196/15448 (PMC7391669; doi:10.2196/15448)
Supplement: Multimedia Appendix 6 [file mhealth_v8i7e15448_app6.docx]

### Sample size calculations for a future RCT

| Table S7. Possible sample size calculations for a future full-scale RCT. | | | | | | | |
| --- | --- | --- | --- | --- | --- | --- | --- |
| HbA1c (mmol/mol), mean difference | Weight (kg), mean difference | Physical activity (steps), mean difference | Effect size (d) | Statistical power | Per-arm sample size | Total sample size with 20% attrition | Time to recruitment (weeks) |
| 0.25 | 1.5 | 359 | 0.1 | 0.8 | 1571 | 3770 | 137 |
|  |  |  |  | 0.9 | 2103 | 5047 | 184 |
| 0.50 | 3.1 | 718 | 0.2 | 0.8 | 394 | 946 | 34 |
|  |  |  |  | 0.9 | 527 | 1265 | 46 |
| 0.76 | 4.6 | 1077 | 0.3 | 0.8 | 176 | 422 | 15 |
|  |  |  |  | 0.9 | 235 | 564 | 21 |
| Note: Statistical significance is set at 0.05 two-tailed. Values are rounded for clarity. The pooled SD for HbA1c was 2.52 mmol/mol. | | | | | | | |
