# Supplementary material for: A Wearable Technology Delivering a Web-Based Diabetes Prevention Program to People at High Risk of Type 2 Diabetes: Randomized Controlled Trial
Source: JMIR Mhealth Uhealth. 2020 Jul 15;8(7):e15448. doi: 10.2196/15448 (PMC7391669; doi:10.2196/15448)
Supplement: Multimedia Appendix 7 [file mhealth_v8i7e15448_app7.pdf]

CONSORT-EHEALTH (V 1.6.1) - Submission/Publication Form

The CONSORT-EHEALTH checklist is intended for authors of randomized trials evaluating web-based and Internet-based applications/interventions, including mobile interventions, electronic games (incl multiplayer games), social media, certain telehealth applications, and other interactive and/or networked electronic applications. Some of the items (e.g. all subitems under item 5 - description of the intervention) may also be applicable for other study designs.

The goal of the CONSORT EHEALTH checklist and guideline is to be
a) a guide for reporting for authors of RCTs,
b) to form a basis for appraisal of an ehealth trial (in terms of validity)

CONSORT-EHEALTH items/subitems are MANDATORY reporting items for studies published in the Journal of Medical Internet Research and other journals / scientific societies endorsing the checklist.

Items numbered 1., 2., 3., 4a., 4b etc are original CONSORT or CONSORT-NPT (non-pharmacologic treatment) items.
Items with Roman numerals (i., ii, iii, iv etc.) are CONSORT-EHEALTH extensions/clarifications.

As the CONSORT-EHEALTH checklist is still considered in a formative stage, we would ask that you also RATE ON A SCALE OF 1-5 how important/useful you feel each item is FOR THE PURPOSE OF THE CHECKLIST and reporting guideline (optional).

Mandatory reporting items are marked with a red *.
In the textboxes, either copy & paste the relevant sections from your manuscript into this form - please include any quotes from your manuscript in QUOTATION MARKS,
or answer directly by providing additional information not in the manuscript, or elaborating on why the item was not relevant for this study.

YOUR ANSWERS WILL BE PUBLISHED AS A SUPPLEMENTARY FILE TO YOUR PUBLICATION IN JMIR AND ARE CONSIDERED PART OF YOUR PUBLICATION (IF ACCEPTED).
Please fill in these questions diligently. Information will not be copyedited, so please use proper spelling and grammar, use correct capitalization, and avoid abbreviations.

DO NOT FORGET TO SAVE AS PDF _AND_ CLICK THE SUBMIT BUTTON SO YOUR ANSWERS ARE IN OUR DATABASE !!!

Citation Suggestion (if you append the pdf as Appendix we suggest to cite this paper in the caption):
Eysenbach G, CONSORT-EHEALTH Group
CONSORT-EHEALTH: Improving and Standardizing Evaluation Reports of Web-based and Mobile Health Interventions
J Med Internet Res 2011;13(4):e126
URL: http://www.jmir.org/2011/4/e126/
doi: 10.2196/jmir.1923
PMID: 22209829

* Required

Your name *

First Last

Emily Staite

Primary Affiliation (short), City, Country *

University of Toronto, Toronto, Canada

King's College London, UK

Your e-mail address *

abc@gmail.com

emily.r.staite@kcl.ac.uk

Title of your manuscript *

Provide the (draft) title of your manuscript.

A randomised controlled trial of a wearable technology delivering an e-diabetes prevention programme in people at high risk of type 2 diabetes: A mixed methods feasibility study

Name of your App/Software/Intervention *

If there is a short and a long/alternate name, write the short name first and add the long name in brackets.

Buddi Ltd

Evaluated Version (if any)

e.g. "V1", "Release 2017-03-01", "Version 2.0.27913"

Your answer

Language(s) *

What language is the intervention/app in? If multiple languages are available, separate by comma (e.g. "English, French")

English

URL of your Intervention Website or App

e.g. a direct link to the mobile app on app in appstore (itunes, Google Play), or URL of the website. If the intervention is a DVD or hardware, you can also link to an Amazon page.

Your answer

URL of an image/screenshot (optional)

Your answer

Accessibility *

Can an enduser access the intervention presently?

- ○ access is free and open
- ○ access only for special usergroups, not open
- ○ access is open to everyone, but requires payment/subscription/in-app purchases
- ◉ app/intervention no longer accessible
- ○ Other:

Primary Medical Indication/Disease/Condition *

e.g. "Stress", "Diabetes", or define the target group in brackets after the condition, e.g. "Autism (Parents of children with)", "Alzheimers (Informal Caregivers of)"

Type 2 Diabetes

Primary Outcomes measured in trial *

comma-separated list of primary outcomes reported in the trial

"The primary aims were to assess 1) the

Secondary/other outcomes

Are there any other outcomes the intervention is expected to affect?

"Our secondary aims were to measure the change in biomedical outcomes, including reducing weight and increasing physical activity, to inform the possible range of effect sizes and obtain outcome variance estimates required for sample size calculations in a full-scale trial. We also conducted focus groups to assess the barriers and facilitators of uptake with participants in both groups."

Recommended "Dose" *

What do the instructions for users say on how often the app should be used?

- (●) Approximately Daily
- ( ) Approximately Weekly
- ( ) Approximately Monthly
- ( ) Approximately Yearly
- ( ) "as needed"
- ( ) Other:

Approx. Percentage of Users (starters) still using the app as recommended after 3 months *

- (selected) unknown / not evaluated
- 0-10%
- 11-20%
- 21-30%
- 31-40%
- 41-50%
- 51-60%
- 61-70%
- 71%-80%
- 81-90%
- 91-100%
- Other:

Overall, was the app/intervention effective? *

- ○ yes: all primary outcomes were significantly better in intervention group vs control
- ○ partly: SOME primary outcomes were significantly better in intervention group vs control
- ○ no statistically significant difference between control and intervention
- ○ potentially harmful: control was significantly better than intervention in one or more outcomes
- ○ inconclusive: more research is needed
- ◉ Other: Feasibility study

Article Preparation Status/Stage *

At which stage in your article preparation are you currently (at the time you fill in this form)

- ○ not submitted yet - in early draft status
- ◉ not submitted yet - in late draft status, just before submission
- ○ submitted to a journal but not reviewed yet
- ○ submitted to a journal and after receiving initial reviewer comments
- ○ submitted to a journal and accepted, but not published yet
- ○ published
- ○ Other:

Journal *

If you already know where you will submit this paper (or if it is already submitted), please provide the journal name (if it is not JMIR, provide the journal name under "other")

- ○ not submitted yet / unclear where I will submit this
- ◉ Journal of Medical Internet Research (JMIR)
- ○ JMIR mHealth and UHealth
- ○ JMIR Serious Games
- ○ JMIR Mental Health
- ○ JMIR Public Health
- ○ JMIR Formative Research
- ○ Other JMIR sister journal
- ○ Other:

Is this a full powered effectiveness trial or a pilot/feasibility trial? *

- ◉ Pilot/feasibility
- ○ Fully powered

Manuscript tracking number *

If this is a JMIR submission, please provide the manuscript tracking number under "other" (The ms tracking number can be found in the submission acknowledgement email, or when you login as author in JMIR. If the paper is already published in JMIR, then the ms tracking number is the four-digit number at the end of the DOI, to be found at the bottom of each published article in JMIR)

- ◉ no ms number (yet) / not (yet) submitted to / published in JMIR
- ○ Other:

TITLE AND ABSTRACT

1a) TITLE: Identification as a randomized trial in the title

1a) Does your paper address CONSORT item 1a? *

I.e does the title contain the phrase "Randomized Controlled Trial"? (if not, explain the reason under "other")

- (•) yes
- ( ) Other:

1a-i) Identify the mode of delivery in the title

Identify the mode of delivery. Preferably use “web-based” and/or “mobile” and/or “electronic game” in the title. Avoid ambiguous terms like “online”, “virtual”, “interactive”. Use “Internet-based” only if Intervention includes non-web-based Internet components (e.g. email), use “computer-based” or “electronic” only if offline products are used. Use “virtual” only in the context of “virtual reality” (3-D worlds). Use “online” only in the context of “online support groups”. Complement or substitute product names with broader terms for the class of products (such as “mobile” or “smart phone” instead of “iphone”), especially if the application runs on different platforms.

	1	2	3	4	5	
subitem not at all important	( )	( )	( )	( )	( )	essential

Does your paper address subitem 1a-i? *

Copy and paste relevant sections from manuscript title (include quotes in quotation marks "like this" to indicate direct quotes from your manuscript), or elaborate on this item by providing additional information not in the ms, or briefly explain why the item is not applicable/relevant for your study

"A randomised controlled trial of a wearable technology delivering an e-diabetes prevention programme in people at high risk of type 2 diabetes: A mixed methods feasibility study"

1a-ii) Non-web-based components or important co-interventions in title

Mention non-web-based components or important co-interventions in title, if any (e.g., “with telephone support”).

	1	2	3	4	5	
subitem not at all important	( )	( )	( )	( )	( )	essential

Does your paper address subitem 1a-ii?

Copy and paste relevant sections from manuscript title (include quotes in quotation marks "like this" to indicate direct quotes from your manuscript), or elaborate on this item by providing additional information not in the ms, or briefly explain why the item is not applicable/relevant for your study

N/A as intervention is not non-web based.

1a-iii) Primary condition or target group in the title

Mention primary condition or target group in the title, if any (e.g., "for children with Type I Diabetes") Example: A Web-based and Mobile Intervention with Telephone Support for Children with Type I Diabetes: Randomized Controlled Trial

	1	2	3	4	5	
subitem not at all important	○	○	○	○	○	essential

Does your paper address subitem 1a-iii? *

Copy and paste relevant sections from manuscript title (include quotes in quotation marks "like this" to indicate direct quotes from your manuscript), or elaborate on this item by providing additional information not in the ms, or briefly explain why the item is not applicable/relevant for your study

"in people at high risk of type 2 diabetes"

1b) ABSTRACT: Structured summary of trial design, methods, results, and conclusions

NPT extension: Description of experimental treatment, comparator, care providers, centers, and blinding status.

1b-i) Key features/functionalities/components of the intervention and comparator in the METHODS section of the ABSTRACT

Mention key features/functionalities/components of the intervention and comparator in the abstract. If possible, also mention theories and principles used for designing the site. Keep in mind the needs of systematic reviewers and indexers by including important synonyms. (Note: Only report in the abstract what the main paper is reporting. If this information is missing from the main body of text, consider adding it)

	1	2	3	4	5	
subitem not at all important	○	○	○	○	○	essential

Does your paper address subitem 1b-i? *

Copy and paste relevant sections from the manuscript abstract (include quotes in quotation marks "like this" to indicate direct quotes from your manuscript), or elaborate on this item by providing additional information not in the ms, or briefly explain why the item is not applicable/relevant for your study

"The intervention integrated a smartphone application delivering an online e-DPP course with text messages incorporating motivational interviewing techniques and biofeedback delivered via a wearable device over a period of 12 months. The control group received the wearable technology and access to the e-DDP, but not the text messaging. As this was a feasibility study, the primary measures were estimating the potential sample size at different stages of the study, from the size of the target study population to the proportion of participants who completed follow-up data collection. We also measured the main outcomes as for a full-scale RCT, namely change in weight and physical activity (PA) at six- and 12-month follow-ups. We conducted a process evaluation, including focus groups with participants who: i) received the intervention, ii) were in the control group, and iii) dropped out of the intervention. The subsequent thematic analysis followed a six-phase process."

1b-ii) Level of human involvement in the METHODS section of the ABSTRACT

Clarify the level of human involvement in the abstract, e.g., use phrases like "fully automated" vs. "therapist/nurse/care provider/physician-assisted" (mention number and expertise of providers involved, if any). (Note: Only report in the abstract what the main paper is reporting. If this information is missing from the main body of text, consider adding it)

	1	2	3	4	5	
subitem not at all important	○	○	○	○	○	essential

Does your paper address subitem 1b-ii?

Copy and paste relevant sections from the manuscript abstract (include quotes in quotation marks "like this" to indicate direct quotes from your manuscript), or elaborate on this item by providing additional information not in the ms, or briefly explain why the item is not applicable/relevant for your study

The level of human involvement included the research team screening patients for eligibility, and assessing patients at baseline, 6 months and 12 months.

1b-iii) Open vs. closed, web-based (self-assessment) vs. face-to-face assessments in the METHODS section of the ABSTRACT

Mention how participants were recruited (online vs. offline), e.g., from an open access website or from a clinic or a closed online user group (closed usergroup trial), and clarify if this was a purely web-based trial, or there were face-to-face components (as part of the intervention or for assessment). Clearly say if outcomes were self-assessed through questionnaires (as common in web-based trials). Note: In traditional offline trials, an open trial (open-label trial) is a type of clinical trial in which both the researchers and participants know which treatment is being administered. To avoid confusion, use “blinded” or “unblinded” to indicated the level of blinding instead of “open”, as “open” in web-based trials usually refers to “open access” (i.e. participants can self-enrol). (Note: Only report in the abstract what the main paper is reporting. If this information is missing from the main body of text, consider adding it)

	1	2	3	4	5	
subitem not at all important	○	○	○	○	○	essential

Does your paper address subitem 1b-iii?

Copy and paste relevant sections from the manuscript abstract (include quotes in quotation marks "like this" to indicate direct quotes from your manuscript), or elaborate on this item by providing additional information not in the ms, or briefly explain why the item is not applicable/relevant for your study

"Patients at high risk of developing type 2 diabetes were identified by the general practitioner (GP) who conducted a search using HbA1c results recorded in the previous 12 months. Participants potentially eligible were then invited for a further screening. Those who had a current HbA1c level of 39–47 mmol/mol were invited to volunteer."

1b-iv) RESULTS section in abstract must contain use data

Report number of participants enrolled/assessed in each group, the use/uptake of the intervention (e.g., attrition/adherence metrics, use over time, number of logins etc.), in addition to primary/secondary outcomes. (Note: Only report in the abstract what the main paper is reporting. If this information is missing from the main body of text, consider adding it)

	1	2	3	4	5	
subitem not at all important	○	○	○	○	○	essential

Does your paper address subitem 1b-iv?

Copy and paste relevant sections from the manuscript abstract (include quotes in quotation marks "like this" to indicate direct quotes from your manuscript), or elaborate on this item by providing additional information not in the ms, or briefly explain why the item is not applicable/relevant for your study

"We enrolled 200 participants: 98 were randomized to the intervention group and 102 to the control group. The follow-up rate was higher in the control group (87 [85.3%]) than the intervention group (69 [70.4%]) for the primary outcomes at 12 months. There was no treatment effect on weight at six months (mean difference=0.15, 95% CI=-0.93 to 1.23) or 12 months (mean difference=0.07 kg, 95% CI=-1.29 to 1.44), or for PA levels at six months (mean difference=-382.90 steps, 95% CI=-860.65 to 94.85) or 12 months (mean difference=92.64 steps, 95% CI=-380.92 to 566.20)."

1b-v) CONCLUSIONS/DISCUSSION in abstract for negative trials

Conclusions/Discussions in abstract for negative trials: Discuss the primary outcome - if the trial is negative (primary outcome not changed), and the intervention was not used, discuss whether negative results are attributable to lack of uptake and discuss reasons. (Note: Only report in the abstract what the main paper is reporting. If this information is missing from the main body of text, consider adding it)

	1	2	3	4	5	
subitem not at all important	○	○	○	○	○	essential

Does your paper address subitem 1b-v?

Copy and paste relevant sections from the manuscript abstract (include quotes in quotation marks "like this" to indicate direct quotes from your manuscript), or elaborate on this item by providing additional information not in the ms, or briefly explain why the item is not applicable/relevant for your study

"We have demonstrated the feasibility of conducting a RCT of an e-DPP. We found that the e-DPP intervention was not effective in improving participants' weight or PA levels. The process evaluation identified several barriers to implementing the intervention."

INTRODUCTION

2a) In INTRODUCTION: Scientific background and explanation of rationale

2a-i) Problem and the type of system/solution

Describe the problem and the type of system/solution that is object of the study: intended as stand-alone intervention vs. incorporated in broader health care program? Intended for a particular patient population? Goals of the intervention, e.g., being more cost-effective to other interventions, replace or complement other solutions? (Note: Details about the intervention are provided in “Methods” under 5)

	1	2	3	4	5	
subitem not at all important	○	○	○	○	○	essential

Does your paper address subitem 2a-i? *

Copy and paste relevant sections from the manuscript (include quotes in quotation marks "like this" to indicate direct quotes from your manuscript), or elaborate on this item by providing additional information not in the ms, or briefly explain why the item is not applicable/relevant for your study

"The prevalence of pre-diabetes is approximately 10% in the UK population [1], with higher rates in adults aged 40 or more years and those of South Asian or African Caribbean ethnicities [2]. Considering the rising prevalence of type 2 diabetes, which accounts for a disproportionate and increasing costs to the individual, society, and healthcare systems globally [3, 4], primary prevention is a current priority.

Landmark randomised controlled trials (RCT) have repeatedly shown that intensive face-to-face diabetes prevention programmes (DPP) are effective in reducing the risk of type 2 diabetes by around 50% [5–7]. The implementation of these landmark studies is expensive, time-consuming for the patient and for health systems, and the uptake is often by those with the lowest risk for type 2 diabetes [8]. One solution is adapting the traditional DPP into an electronic format (e-DPP) using wearable technologies and online programmes. These are increasingly available from commercial providers at a low cost to the patient and/or health commissioners, but the evidence of their effectiveness in reducing the risk of type 2 diabetes or causing harm is limited [9, 10]."

2a-ii) Scientific background, rationale: What is known about the (type of) system

Scientific background, rationale: What is known about the (type of) system that is the object of the study (be sure to discuss the use of similar systems for other conditions/diagnoses, if appropiate), motivation for the study, i.e. what are the reasons for and what is the context for this specific study, from which stakeholder viewpoint is the study performed, potential impact of findings [2]. Briefly justify the choice of the comparator.

	1	2	3	4	5	
subitem not at all important	○	○	○	○	○	essential

Does your paper address subitem 2a-ii? *

Copy and paste relevant sections from the manuscript (include quotes in quotation marks "like this" to indicate direct quotes from your manuscript), or elaborate on this item by providing additional information not in the ms, or briefly explain why the item is not applicable/relevant for your study

"Components of behavioural change techniques considered to be most effective in improving diet and physical activity are based on self-regulatory behaviours, such as goal setting, self-monitoring, giving feedback, utilising social support, and motivational interviewing (MI) [14, 15]. Interventions based on a psychological theory (e.g., the theory of planned behaviour) are considered to be more effective and have better outcomes in high-risk populations [16], although the minimum threshold of intensity, such as number of sessions or messages or duration of the intervention, is not known [14, 17, 18]."

2b) In INTRODUCTION: Specific objectives or hypotheses

Does your paper address CONSORT subitem 2b? *

Copy and paste relevant sections from the manuscript (include quotes in quotation marks "like this" to indicate direct quotes from your manuscript), or elaborate on this item by providing additional information not in the ms, or briefly explain why the item is not applicable/relevant for your study

"This RCT aims to test the feasibility of an e-DPP, consisting of a wearable technology recording physical activity, integrated with text messaging based on MI techniques and lifestyle education delivered via a smartphone application, over 12 months in participants with pre-diabetes. The primary aims were to assess: 1) the potential size of the study population; 2) the proportion who consent to be screened for eligibility; 3) the proportion of those who were screened who were eligible, consented, and randomized; 4) the proportion of those randomised who completed the intervention; and 5) the proportion who completed the six- and 12-month follow-ups.

Our secondary aims were to measure the change in biomedical outcomes, including reducing weight and increasing physical activity, to inform the possible range of effect sizes and obtain outcome variance estimates required for sample size calculations in a full-scale trial. We also conducted focus groups to assess the barriers and facilitators of uptake with participants in both groups."

METHODS

3a) Description of trial design (such as parallel, factorial) including allocation ratio

Does your paper address CONSORT subitem 3a? *

Copy and paste relevant sections from the manuscript (include quotes in quotation marks "like this" to indicate direct quotes from your manuscript), or elaborate on this item by providing additional information not in the ms, or briefly explain why the item is not applicable/relevant for your study

"This was a two-arm, parallel, single-blind RCT over 12 months."

3b) Important changes to methods after trial commencement (such as eligibility criteria), with reasons

Does your paper address CONSORT subitem 3b? *

Copy and paste relevant sections from the manuscript (include quotes in quotation marks "like this" to indicate direct quotes from your manuscript), or elaborate on this item by providing additional information not in the ms, or briefly explain why the item is not applicable/relevant for your study

No changes to methods after trail commencement.

3b-i) Bug fixes, Downtimes, Content Changes

Bug fixes, Downtimes, Content Changes: ehealth systems are often dynamic systems. A description of changes to methods therefore also includes important changes made on the intervention or comparator during the trial (e.g., major bug fixes or changes in the functionality or content) (5-iii) and other “unexpected events” that may have influenced study design such as staff changes, system failures/downtimes, etc. [2].

	1	2	3	4	5	
subitem not at all important	○	○	○	○	○	essential

Does your paper address subitem 3b-i?

Copy and paste relevant sections from the manuscript (include quotes in quotation marks "like this" to indicate direct quotes from your manuscript), or elaborate on this item by providing additional information not in the ms, or briefly explain why the item is not applicable/relevant for your study

No changes to methods after trail commencement.

4a) Eligibility criteria for participants

Does your paper address CONSORT subitem 4a? *

Copy and paste relevant sections from the manuscript (include quotes in quotation marks "like this" to indicate direct quotes from your manuscript), or elaborate on this item by providing additional information not in the ms, or briefly explain why the item is not applicable/relevant for your study

In addition to having a HbA1c level of 39–47 mmol/mol, "the inclusion criteria were: adults aged between 18-65 years; body mass index (BMI) ≥ 25 kg/m² (≥ 23 kg/m² if of Asian ethnicity) [19]); permanent residents in Lambeth, Southwark, or Lewisham; owning a smartphone (iPhone or Android models only) defined as logging on at least once/day to the internet; being fluent in conversational English; and ambulatory.

The exclusion criteria were: known diabetes (not including past history of gestational diabetes), pregnancy, planning pregnancy or lactating during the duration of the study; severe mental illness (severe depression with suicidal ideation, psychosis, bipolar affective disorder, dementia, learning difficulties, substance problem use or dependence); severe physical disability (e.g., that would prevent any increased uptake of physical exercise); advanced active disease, such as cancer or heart failure; any other condition which requires glucose-altering drugs; BMI ≥50 kg/m²; and current participation in a weight loss programme or DPP. Where in doubt, we sought GP confirmation of eligibility."

4a-i) Computer / Internet literacy

Computer / Internet literacy is often an implicit “de facto” eligibility criterion - this should be explicitly clarified.

	1	2	3	4	5	
subitem not at all important	○	○	○	○	○	essential

Does your paper address subitem 4a-i?

Copy and paste relevant sections from the manuscript (include quotes in quotation marks "like this" to indicate direct quotes from your manuscript), or elaborate on this item by providing additional information not in the ms, or briefly explain why the item is not applicable/relevant for your study

Participants must "own a smartphone (iPhone or Android models only) defined as logging on at least once/day to the internet; be fluent in conversational English"

4a-ii) Open vs. closed, web-based vs. face-to-face assessments:

Open vs. closed, web-based vs. face-to-face assessments: Mention how participants were recruited (online vs. offline), e.g., from an open access website or from a clinic, and clarify if this was a purely web-based trial, or there were face-to-face components (as part of the intervention or for assessment), i.e., to what degree got the study team to know the participant. In online-only trials, clarify if participants were quasi-anonymous and whether having multiple identities was possible or whether technical or logistical measures (e.g., cookies, email confirmation, phone calls) were used to detect/prevent these.

	1	2	3	4	5	
subitem not at all important	○	○	○	○	○	essential

Does your paper address subitem 4a-ii? *

Copy and paste relevant sections from the manuscript (include quotes in quotation marks "like this" to indicate direct quotes from your manuscript), or elaborate on this item by providing additional information not in the ms, or briefly explain why the item is not applicable/relevant for your study

"Patients with an HbA1c of 39–47 mmol/mol were defined as having pre-diabetes state according to current international criteria [21]. They were identified in a two-stage process. First, patients at high risk of developing type 2 diabetes were identified by the general practitioner (GP) who conducted a search using HbA1c results recorded in the previous 12 months and invited those potentially eligible (HbA1c level of 39–47 mmol/mol) for further screening. Second, those patients who attended the screening and had a current HbA1c level of 39–47 mmol/mol were invited to volunteer."

Patients were assessed face-to-face by the research team at baseline, 6 months and 12 months. The app and wristband recorded physical activity over 12 months.

4a-iii) Information giving during recruitment

Information given during recruitment. Specify how participants were briefed for recruitment and in the informed consent procedures (e.g., publish the informed consent documentation as appendix, see also item X26), as this information may have an effect on user self-selection, user expectation and may also bias results.

	1	2	3	4	5	
subitem not at all important	○	○	○	○	○	essential

Does your paper address subitem 4a-iii?

Copy and paste relevant sections from the manuscript (include quotes in quotation marks "like this" to indicate direct quotes from your manuscript), or elaborate on this item by providing additional information not in the ms, or briefly explain why the item is not applicable/relevant for your study

Information for participants were provided in an invitation letter, participant information sheet and consent form.

4b) Settings and locations where the data were collected

Does your paper address CONSORT subitem 4b? *

Copy and paste relevant sections from the manuscript (include quotes in quotation marks "like this" to indicate direct quotes from your manuscript), or elaborate on this item by providing additional information not in the ms, or briefly explain why the item is not applicable/relevant for your study

"Physical activity was measured objectively using the wearable technology (wristband). Physical activity (number of steps per day) was recorded continuously by the wristband. Baseline physical activity was the mean step count of the first seven days of wearing the wristband (starting from the baseline appointment). Days with no recorded steps were removed before calculating each participant's mean step count."

The following data was collected by the research team in the baseline/6month/12month appointments with participants at their GP surgery:

"We collected sociodemographic data, including age, gender, postcode of residence, employment status, educational level, and self-reported ethnicity. Based on the participant's postcode, we determined their Indices of Multiple Deprivation (IMD) 2015 rank, which indicates the relative level of socioeconomic deprivation in their area [22]."

Self-reported physical activity was assessed using the International Physical Activity Questionnaire (IPAQ) [23], The following biomedical data were collected: HbA1c level (mmol/mol) and lipids (total cholesterol, high- and low-density lipoproteins (HDL and LDL), and fasting triglycerides (mmol/L)). Weight was measured in light clothing, without shoes, to 0.01 kg. Height was measured to 0.1 cm using stadiometers. Weight and height measurements were used to calculate BMI (kg/m2). Waist circumference (cm) was measured horizontally halfway between the lowest rib and the upper prominence of the pelvis using a non-extensible steel tape against the bare abdomen. Hip circumference was also measured to calculate waist-to-hip ratio. BP and resting heart rate were measured with digital Omron BP monitors (Omron, Kyoto, Japan) using standardised procedures of the average of two readings taken one minute apart while seated.

Depressive symptoms were self-reported using the Patient Health Questionnaire-9 (PHQ-9) [24]. Readiness to change was measured using the University of Rhode Island Change Assessment Scale (URICA) [25], adapted for the current study to ask specifically about participants' readiness to change dietary and activity behaviours with regards to their health. Self-efficacy was measured using the Self-Efficacy for Exercise (SEE) Scale. We collected data on smoking status and, if current, how many cigarettes per day. Alcohol intake was measured using the Alcohol Use Disorders Identification Test (AUDIT) [27]."

4b-i) Report if outcomes were (self-)assessed through online questionnaires

Clearly report if outcomes were (self-)assessed through online questionnaires (as common in web-based trials) or otherwise.

	1	2	3	4	5	
subitem not at all important	○	○	○	○	○	essential

Does your paper address subitem 4b-i? *

Copy and paste relevant sections from the manuscript (include quotes in quotation marks "like this" to indicate direct quotes from your manuscript), or elaborate on this item by providing additional information not in the ms, or briefly explain why the item is not applicable/relevant for your study

Outcomes were not assessed through online questionnaires.

4b-ii) Report how institutional affiliations are displayed

Report how institutional affiliations are displayed to potential participants [on ehealth media], as affiliations with prestigious hospitals or universities may affect volunteer rates, use, and reactions with regards to an intervention.(Not a required item – describe only if this may bias results)

	1	2	3	4	5	
subitem not at all important	○	○	○	○	○	essential

Does your paper address subitem 4b-ii?

Copy and paste relevant sections from the manuscript (include quotes in quotation marks "like this" to indicate direct quotes from your manuscript), or elaborate on this item by providing additional information not in the ms, or briefly explain why the item is not applicable/relevant for your study

Institutional affiliations ("King's College London") were displayed on the letterhead of the participant information sheet and consent form. The smartphone application delivering an online e-DPP course with text messages did not include institutional affiliations.

5) The interventions for each group with sufficient details to allow replication, including how and when they were actually administered

5-i) Mention names, credential, affiliations of the developers, sponsors, and owners

Mention names, credential, affiliations of the developers, sponsors, and owners [6] (if authors/evaluators are owners or developer of the software, this needs to be declared in a “Conflict of interest” section or mentioned elsewhere in the manuscript).

	1	2	3	4	5	
subitem not at all important	○	○	○	○	○	essential

Does your paper address subitem 5-i?

Copy and paste relevant sections from the manuscript (include quotes in quotation marks "like this" to indicate direct quotes from your manuscript), or elaborate on this item by providing additional information not in the ms, or briefly explain why the item is not applicable/relevant for your study

Buddi Ltd provided the application and wristband.

5-ii) Describe the history/development process

Describe the history/development process of the application and previous formative evaluations (e.g., focus groups, usability testing), as these will have an impact on adoption/use rates and help with interpreting results.

	1	2	3	4	5	
subitem not at all important	○	○	○	○	○	essential

Does your paper address subitem 5-ii?

Copy and paste relevant sections from the manuscript (include quotes in quotation marks "like this" to indicate direct quotes from your manuscript), or elaborate on this item by providing additional information not in the ms, or briefly explain why the item is not applicable/relevant for your study

Buddi Ltd was founded in 2005 and develops mobile technologies for vulnerable individuals. Buddi provides technologies to support service users across more than 80% of UK local authorities, as well as to Government customers around the world.

5-iii) Revisions and updating

Revisions and updating. Clearly mention the date and/or version number of the application/intervention (and comparator, if applicable) evaluated, or describe whether the intervention underwent major changes during the evaluation process, or whether the development and/or content was “frozen” during the trial. Describe dynamic components such as news feeds or changing content which may have an impact on the replicability of the intervention (for unexpected events see item 3b).

	1	2	3	4	5	
subitem not at all important	○	○	○	○	○	essential

Does your paper address subitem 5-iii?

Copy and paste relevant sections from the manuscript (include quotes in quotation marks "like this" to indicate direct quotes from your manuscript), or elaborate on this item by providing additional information not in the ms, or briefly explain why the item is not applicable/relevant for your study

The development and/or content was not “frozen” during the trial.

5-iv) Quality assurance methods

Provide information on quality assurance methods to ensure accuracy and quality of information provided [1], if applicable.

	1	2	3	4	5	
subitem not at all important	○	○	○	○	○	essential

Does your paper address subitem 5-iv?

Copy and paste relevant sections from the manuscript (include quotes in quotation marks "like this" to indicate direct quotes from your manuscript), or elaborate on this item by providing additional information not in the ms, or briefly explain why the item is not applicable/relevant for your study

All members of the research team were trained on how to administer the questionnaires in the baseline, 6 month and 12 month follow up appointments. All questionnaires selected have been consistently used in research due to their high levels of reliability, validity etc.

5-v) Ensure replicability by publishing the source code, and/or providing screenshots/screen-capture video, and/or providing flowcharts of the algorithms used

Ensure replicability by publishing the source code, and/or providing screenshots/screen-capture video, and/or providing flowcharts of the algorithms used. Replicability (i.e., other researchers should in principle be able to replicate the study) is a hallmark of scientific reporting.

	1	2	3	4	5	
subitem not at all important	○	○	○	○	○	essential

Does your paper address subitem 5-v?

Copy and paste relevant sections from the manuscript (include quotes in quotation marks "like this" to indicate direct quotes from your manuscript), or elaborate on this item by providing additional information not in the ms, or briefly explain why the item is not applicable/relevant for your study

The methods section (including design, setting, participants, baseline measures, intervention, outcome measures, sample size, randomisation and allocation concealment, statistical analysis plan, qualitative analysis) has been described allowing replication of study.

5-vi) Digital preservation

Digital preservation: Provide the URL of the application, but as the intervention is likely to change or disappear over the course of the years; also make sure the intervention is archived (Internet Archive, webcitation.org, and/or publishing the source code or screenshots/videos alongside the article). As pages behind login screens cannot be archived, consider creating demo pages which are accessible without login.

	1	2	3	4	5	
subitem not at all important	○	○	○	○	○	essential

Does your paper address subitem 5-vi?

Copy and paste relevant sections from the manuscript (include quotes in quotation marks "like this" to indicate direct quotes from your manuscript), or elaborate on this item by providing additional information not in the ms, or briefly explain why the item is not applicable/relevant for your study

Intervention needs to be archived.

5-vii) Access

Access: Describe how participants accessed the application, in what setting/context, if they had to pay (or were paid) or not, whether they had to be a member of specific group. If known, describe how participants obtained “access to the platform and Internet” [1]. To ensure access for editors/reviewers/readers, consider to provide a “backdoor” login account or demo mode for reviewers/readers to explore the application (also important for archiving purposes, see vi).

	1	2	3	4	5	
subitem not at all important	○	○	○	○	○	essential

Does your paper address subitem 5-vii? *

Copy and paste relevant sections from the manuscript (include quotes in quotation marks "like this" to indicate direct quotes from your manuscript), or elaborate on this item by providing additional information not in the ms, or briefly explain why the item is not applicable/relevant for your study

"The intervention integrated a smartphone application delivering an online e-DPP course with text messages incorporating motivational interviewing techniques and biofeedback delivered via a wearable device over a period of 12 months. The control group received the wearable technology and access to the e-DDP, but not the text messaging." Participants accessed the application via their mobile phone. Participants were voluntary (i.e. not paid to take part in the study).

5-viii) Mode of delivery, features/functionalities/components of the intervention and comparator, and the theoretical framework

Describe mode of delivery, features/functionalities/components of the intervention and comparator, and the theoretical framework [6] used to design them (instructional strategy [1], behaviour change techniques, persuasive features, etc., see e.g., [7, 8] for terminology). This includes an in-depth description of the content (including where it is coming from and who developed it) [1],” whether [and how] it is tailored to individual circumstances and allows users to track their progress and receive feedback” [6]. This also includes a description of communication delivery channels and – if computer-mediated communication is a component – whether communication was synchronous or asynchronous [6]. It also includes information on presentation strategies [1], including page design principles, average amount of text on pages, presence of hyperlinks to other resources, etc. [1].

	1	2	3	4	5	
subitem not at all important	○	○	○	○	○	essential

Does your paper address subitem 5-viii? *

Copy and paste relevant sections from the manuscript (include quotes in quotation marks "like this" to indicate direct quotes from your manuscript), or elaborate on this item by providing additional information not in the ms, or briefly explain why the item is not applicable/relevant for your study

"Wearable Technology
All participants were issued with a wristband (manufactured and provided by Buddi Ltd, Rickmansworth), a charger for it, and instructions for operating the wristband and downloading the associated study-specific smartphone application. Participants were instructed to wirelessly connect the Buddi wristband with their smartphone via Bluetooth and to maintain the connection to facilitate the transfer of data captured by the Buddi wristband to the participants' smartphones. This allowed participants to track their activity in close to real-time via the smartphone application, as well as review past activity. If any technical issues arose, participants were able to contact a researcher (who was not blind to intervention allocation) for technical support and any faulty devices were replaced.

Online Education
We timetabled and delivered 22 online sessions over 12 months targeting diet, physical activity, and mental resilience. The curriculum was based on the newly-developed Centers for Disease Control and Prevention (CDC) PreventT2 curriculum and handouts [28], which is itself the implementation version of the original landmark DPP studies [5, 29, 30]. The online sessions were available through the smartphone application, with portable document format (PDF) transcripts available to download for each session.
Intervention Arm
The intervention was based on the theory of planned behaviour which states that in order to change behaviour, people need to form an intention [16]. Intention formation is influenced by three constructs: expected value or positive attitude (people see the value in making the change); subjective norm (significant others and peers also value the change) and self-efficacy (people believe they are capable of making the change).

Our intervention had the following components.
1. Online education as detailed above.
2. Biofeedback; each participant received message summarising their recorded step count.
3. Motivational text messaging: we generated and delivered text messages using principles and techniques from MI to support participants in forming healthy intentions, encourage self-monitoring of lifestyle behaviours, and promote social support [15]. MI is normally a face-to-face collaborative conversation style for strengthening a person's motivation, belief, and commitment to change; we adapted this principle for a virtual setting. The

contents of the automated messages were temporally coordinated with the contents of the educational programme. Participants received between 3-5 messages/day during the working week for 12 months. There were 4 types of messages as follows:

a) Messages welcoming and introducing each new module of the e-DPP (approximately every 2 weeks).
b) Messages targeting lifestyle behaviours: this consisted of messages encouraging 'how' to make lifestyle changes (self-efficacy) followed by 'why' messages (expected value of change to the patient) as reinforcement. The content was coordinated to mirror the contents of the educational programme.
c) Messages giving feedback on the biodata (i.e., the activity data received from the wearable technology on step count); the content was based on the level of activity designed to reinforce or encourage an increase in activity levels.
d) Responsive messages were sent if participants pro-actively selected the following on the app: 'achieve' when they felt they had reached a goal; 'crave' when they were thinking about pursuing an unhealthy behaviour (e.g., eating a high calorie food or avoiding their exercise regime) but had not acted upon it; or 'lapse' when they had acted upon their cravings and need support to re-engage with their good intentions. Participants also had the opportunity to record their achievements within the application.

Control Arm
The control group was provided with the Buddi wristband for the duration of the study and could access their activity data and online education material via the smartphone application. They received an automated message informing them when the next educational session was available (i.e., 22 messages in total), but no other messages."

5-ix) Describe use parameters

Describe use parameters (e.g., intended "doses" and optimal timing for use). Clarify what instructions or recommendations were given to the user, e.g., regarding timing, frequency, heaviness of use, if any, or was the intervention used ad libitum.

	1	2	3	4	5	
subitem not at all important	○	○	○	○	○	essential

Does your paper address subitem 5-ix?

Copy and paste relevant sections from the manuscript (include quotes in quotation marks "like this" to indicate direct quotes from your manuscript), or elaborate on this item by providing additional information not in the ms, or briefly explain why the item is not applicable/relevant for your study

See above.

5-x) Clarify the level of human involvement

Clarify the level of **human inv**olvement (care providers or health professionals, also technical assistance) in the e-intervention or as co-intervention (detail number and expertise of professionals involved, if any, as well as "type of assistance offered, the timing and frequency of the support, how it is initiated, and the medium by which the assistance is delivered". It may be necessary to distinguish between the level of **human inv**olvement required for the trial, and the level of **human inv**olvement required for a routine application outside of a RCT setting (discuss under item 21 – generalizability).

	1	2	3	4	5	
subitem not at all important	○	○	○	○	○	essential

Does your paper address subitem 5-x?

Copy and paste relevant sections from the manuscript (include quotes in quotation marks "like this" to indicate direct quotes from your manuscript), or elaborate on this item by providing additional information not in the ms, or briefly explain why the item is not applicable/relevant for your study

The level of human involvement included the research team screening patients for eligibility, and assessing patients at baseline, 6 months and 12 months. The research team also gave participants technical assistance/troubleshooting advice over the telephone. The research team would seek advice from the technical team at Buddi when needed.

5-xi) Report any prompts/reminders used

Report any prompts/reminders used: Clarify if there were prompts (letters, emails, phone calls, SMS) to use the application, what triggered them, frequency etc. It may be necessary to distinguish between the level of prompts/reminders required for the trial, and the level of prompts/reminders for a routine application outside of a RCT setting (discuss under item 21 – generalizability).

	1	2	3	4	5	
subitem not at all important	○	○	○	○	○	essential

Does your paper address subitem 5-xi? *

Copy and paste relevant sections from the manuscript (include quotes in quotation marks "like this" to indicate direct quotes from your manuscript), or elaborate on this item by providing additional information not in the ms, or briefly explain why the item is not applicable/relevant for your study

No prompts/reminders were used.

5-xii) Describe any co-interventions (incl. training/support)

Describe any co-interventions (incl. training/support): Clearly state any interventions that are provided in addition to the targeted eHealth intervention, as ehealth intervention may not be designed as stand-alone intervention. This includes training sessions and support [1]. It may be necessary to distinguish between the level of training required for the trial, and the level of training for a routine application outside of a RCT setting (discuss under item 21 – generalizability.

	1	2	3	4	5	
subitem not at all important	○	○	○	○	○	essential

Does your paper address subitem 5-xii? *

Copy and paste relevant sections from the manuscript (include quotes in quotation marks "like this" to indicate direct quotes from your manuscript), or elaborate on this item by providing additional information not in the ms, or briefly explain why the item is not applicable/relevant for your study

There were no interventions in addition to the targeted eHealth intervention.

6a) Completely defined pre-specified primary and secondary outcome measures, including how and when they were assessed

Does your paper address CONSORT subitem 6a? *

Copy and paste relevant sections from the manuscript (include quotes in quotation marks "like this" to indicate direct quotes from your manuscript), or elaborate on this item by providing additional information not in the ms, or briefly explain why the item is not applicable/relevant for your study

"The primary outcome measures were change in weight (kg) and physical activity (mean steps per day) from baseline to 12 months, with an interim measure at six months. Follow-up physical activity was the mean step count of the seven days of wear leading up to and including day of the follow-up appointment."

"Weight was measured in light clothing, without shoes, to 0.01 kg."

"Physical activity was measured objectively using the wearable technology (a wristband, see below). Physical activity (number of steps per day) was recorded continuously by the wristband. Baseline physical activity was the mean step count of the first seven days of wearing the wristband (starting from the baseline appointment). Days with no recorded steps were removed before calculating each participant's mean step count (see Appendix for number of days used in calculating step counts)."

"The secondary outcomes were change in HbA1c, waist circumference, waist:hip ratio, lipid levels, and systolic and diastolic blood pressure (BP). HbA1c was analysed as a continuous and categorical variable, with the following categories: normal (<42mmol/mol), pre-diabetes (42-47mmol/mol) and diabetes (>47mmol/mol). We collected interim HbA1c levels and BP at six months, with the other secondary outcomes collected at 12 months."

"Waist circumference (cm) was measured horizontally halfway between the lowest rib and the upper prominence of the pelvis using a non-extensible steel tape against the bare abdomen. Hip circumference was also measured to calculate waist-to-hip ratio. BP and resting heart rate were measured with digital Omron BP monitors (Omron, Kyoto, Japan) using standardised procedures of the average of two readings taken one minute apart while seated."

6a-i) Online questionnaires: describe if they were validated for online use and apply CHERRIES items to describe how the questionnaires were designed/deployed

If outcomes were obtained through online questionnaires, describe if they were validated for online use and apply CHERRIES items to describe how the questionnaires were designed/deployed [9].

	1	2	3	4	5	
subitem not at all important	○	○	○	○	○	essential

Does your paper address subitem 6a-i?

Copy and paste relevant sections from manuscript text

N/A. Outcomes were not obtained through online questionnaires.

6a-ii) Describe whether and how “use” (including intensity of use/dosage) was defined/measured/monitored

Describe whether and how “use” (including intensity of use/dosage) was defined/measured/monitored (logins, logfile analysis, etc.). Use/adoption metrics are important process outcomes that should be reported in any ehealth trial.

	1	2	3	4	5	
subitem not at all important	○	○	○	○	○	essential

Does your paper address subitem 6a-ii?

Copy and paste relevant sections from manuscript text

"Adherence to the intervention was calculated by determining the proportion of days that a participant used the smartphone application and had the wristband connected to it for at least one hour per day. We considered a threshold of ≥14% of days that the participant had their wristband and it was registered with Buddi’s system with at least one hour of usage, from baseline to the participant’s last follow-up or withdrawal, as being minimally adherent to the intervention. This threshold suggests that the participant accessed the application at least once per week, used the application long enough to review their activity and the educational material, and yet accounted for any prolonged technical issues with the application or wristband. Adherence was defined the same for the intervention and control arms."

6a-iii) Describe whether, how, and when qualitative feedback from participants was obtained

Describe whether, how, and when qualitative feedback from participants was obtained (e.g., through emails, feedback forms, interviews, focus groups).

	1	2	3	4	5	
subitem not at all important	○	○	○	○	○	essential

Does your paper address subitem 6a-iii?

Copy and paste relevant sections from manuscript text

"Three focus groups with participants who: i) received the intervention, ii) were in the control group, and iii) dropped out of the intervention, were conducted in July 2018.

At 12 month follow-up appointments participants were offered the opportunity to be contacted (after completion of all follow-up appointments) regarding focus groups. An equal number of males and females who agreed to be contacted were randomly selected and invited to be part of the focus groups. The first six participants of each group (Intervention/Control/Dropped out) were sent a letter confirmation. Those that responded after the group's capacity was full were put on a waiting list in case confirmed participants dropped out. Focus groups were audio-recorded and transcribed verbatim."

6b) Any changes to trial outcomes after the trial commenced, with reasons

Does your paper address CONSORT subitem 6b? *

Copy and paste relevant sections from the manuscript (include quotes in quotation marks "like this" to indicate direct quotes from your manuscript), or elaborate on this item by providing additional information not in the ms, or briefly explain why the item is not applicable/relevant for your study

There were no changes to trial outcomes after the trial commenced.

7a) How sample size was determined

NPT: When applicable, details of whether and how the clustering by care provides or centers was addressed

7a-i) Describe whether and how expected attrition was taken into account when calculating the sample size

Describe whether and how expected attrition was taken into account when calculating the sample size.

	1	2	3	4	5	
subitem not at all important	○	○	○	○	○	essential

Does your paper address subitem 7a-i?

Copy and paste relevant sections from manuscript title (include quotes in quotation marks "like this" to indicate direct quotes from your manuscript), or elaborate on this item by providing additional information not in the ms, or briefly explain why the item is not applicable/relevant for your study

"The primary endpoints of this feasibility trial were factors that would inform a successful full-scale trial conduct, rather than measures of intervention effects. We aimed for 100 participants per arm as this was a large enough sample to inform the practicalities of delivering the intervention, recruitment, uptake, and attrition."

7b) When applicable, explanation of any interim analyses and stopping guidelines

Does your paper address CONSORT subitem 7b? *

Copy and paste relevant sections from the manuscript (include quotes in quotation marks "like this" to indicate direct quotes from your manuscript), or elaborate on this item by providing additional information not in the ms, or briefly explain why the item is not applicable/relevant for your study

N/A. No interim analyses.

8a) Method used to generate the random allocation sequence

NPT: When applicable, how care providers were allocated to each trial group

Does your paper address CONSORT subitem 8a? *

Copy and paste relevant sections from the manuscript (include quotes in quotation marks "like this" to indicate direct quotes from your manuscript), or elaborate on this item by providing additional information not in the ms, or briefly explain why the item is not applicable/relevant for your study

"Randomisation of participants was conducted by the data manager from an independent Clinical Trials Unit using computer-generated randomisation blocks of random sizes and stratified by surgery in a 1:1 ratio. Allocation concealment of the randomisation list was held in a password-locked computer. This was an open-label study, but outcome assessors, laboratory technicians, and researchers entering and scoring the data were blind to patients' allocations."

8b) Type of randomisation; details of any restriction (such as blocking and block size)

Does your paper address CONSORT subitem 8b? *

Copy and paste relevant sections from the manuscript (include quotes in quotation marks "like this" to indicate direct quotes from your manuscript), or elaborate on this item by providing additional information not in the ms, or briefly explain why the item is not applicable/relevant for your study

See 8a (above).

9) Mechanism used to implement the random allocation sequence (such as sequentially numbered containers), describing any steps taken to conceal the sequence until interventions were assigned

Does your paper address CONSORT subitem 9? *

Copy and paste relevant sections from the manuscript (include quotes in quotation marks "like this" to indicate direct quotes from your manuscript), or elaborate on this item by providing additional information not in the ms, or briefly explain why the item is not applicable/relevant for your study

See 8a (above).

10) Who generated the random allocation sequence, who enrolled participants, and who assigned participants to interventions

Does your paper address CONSORT subitem 10? *

Copy and paste relevant sections from the manuscript (include quotes in quotation marks "like this" to indicate direct quotes from your manuscript), or elaborate on this item by providing additional information not in the ms, or briefly explain why the item is not applicable/relevant for your study

See 8a (above).

11a) If done, who was blinded after assignment to interventions (for example, participants, care providers, those assessing outcomes) and how

NPT: Whether or not administering co-interventions were blinded to group assignment

11a-i) Specify who was blinded, and who wasn't

Specify who was blinded, and who wasn't. Usually, in web-based trials it is not possible to blind the participants [1, 3] (this should be clearly acknowledged), but it may be possible to blind outcome assessors, those doing data analysis or those administering co-interventions (if any).

	1	2	3	4	5	
subitem not at all important	○	○	○	○	○	essential

Does your paper address subitem 11a-i? *

Copy and paste relevant sections from the manuscript (include quotes in quotation marks "like this" to indicate direct quotes from your manuscript), or elaborate on this item by providing additional information not in the ms, or briefly explain why the item is not applicable/relevant for your study

"If any technical issues arose, participants were able to contact a researcher (who was not blind to intervention allocation) for technical support and any faulty devices were replaced". The rest of the research team were blinded. Participants were not blinded as they knew they were going to be randomly allocated to the intervention arm (where they would receive messages) or the control arm (where they would not receive messages).

11a-ii) Discuss e.g., whether participants knew which intervention was the “intervention of interest” and which one was the “comparator”

Informed consent procedures (4a-ii) can create biases and certain expectations - discuss e.g., whether participants knew which intervention was the “intervention of interest” and which one was the “comparator”.

	1	2	3	4	5	
subitem not at all important	○	○	○	○	○	essential

Does your paper address subitem 11a-ii?

Copy and paste relevant sections from the manuscript (include quotes in quotation marks "like this" to indicate direct quotes from your manuscript), or elaborate on this item by providing additional information not in the ms, or briefly explain why the item is not applicable/relevant for your study

Participants knew that one group (intervention) would receive messages and one group (control) would not.

11b) If relevant, description of the similarity of interventions

(this item is usually not relevant for ehealth trials as it refers to similarity of a placebo or sham intervention to a active medication/intervention)

Does your paper address CONSORT subitem 11b? *

Copy and paste relevant sections from the manuscript (include quotes in quotation marks "like this" to indicate direct quotes from your manuscript), or elaborate on this item by providing additional information not in the ms, or briefly explain why the item is not applicable/relevant for your study

N/A. No placebo, sham intervention or medication involved in this trial.

12a) Statistical methods used to compare groups for primary and secondary outcomes

NPT: When applicable, details of whether and how the clustering by care providers or centers was addressed

Does your paper address CONSORT subitem 12a? *

Copy and paste relevant sections from the manuscript (include quotes in quotation marks "like this" to indicate direct quotes from your manuscript), or elaborate on this item by providing additional information not in the ms, or briefly explain why the item is not applicable/relevant for your study

"Analysis and reporting were in line with the Consolidated Standards of Reporting Trials (CONSORT) guidelines [31], including its extensions for pilot and feasibility trials, with primary analyses being on an intention-to-treat basis and a 0.05 two-sided significance level. Statistical analyses are mainly descriptive, aiming to provide estimates of key feasibility parameters and to inform power calculations for a future definitive trial. A description of the sample is presented using means and standard deviations (SD) for continuous data and frequencies and proportions for categorical variables. Reasons for non-adherence were examined where information is available. Descriptive sub-analyses were used to explore participation rates among participants based on ethnicity, education level, IMD 2015 score, BMI, depressive symptoms, readiness to change, and self-efficacy. In addition, completion rates for demographic and clinical measures were assessed at baseline and follow-up. [...] We compared baseline characteristics of: a) those who were eligible who did and not consent; and b) those who did and did not provide 12-month follow-up data for the primary outcome

The purpose of the statistical analysis was to test the statistical parameters needed to be considered for a full-scale RCT. The differences in treatment effect for the primary and secondary outcomes between the arms at six- and 12-month follow-ups were analysed using ANCOVA-based, linear mixed-effects models with pre-randomisation values as a covariate [32]. STATA's mixed command was used for estimation. The fixed parts of the model were: the interaction 'treatment group by time' and the pre-randomisation value of the respective outcome. 'Time' was entered as a categorical variable to avoid making parametric assumptions of the outcome values over time. For secondary outcomes measured at 12-month follow-up only, an ANCOVA model was used, covarying for pre-randomisation values.

We conducted a second post hoc sensitivity analysis where weight and the secondary outcomes were re-analysed after excluding participants with missing PA outcome data. While follow-up rates were relatively high at six and 12 months, we observed that a significant proportion of participants did not have PA (i.e., step count) data recorded by their wristband at follow-up. Thus, we investigated whether there were any group differences in the outcomes in the sub-group of participants with recorded PA data.

We conducted a post hoc comparison of participants' baseline characteristics for those that showed any improvements in either of the two primary outcomes

at 12-month follow-up. We used Student’s t-tests and □2-tests to compare continuous and categorical variables, respectively.

We examined the associations between participants’ self-reported activity levels (IPAQ scores) and those objectively measured by the wristband (mean daily step count) by time point and trial arm.

A process evaluation was conducted to identify, describe, and, where appropriate, quantify, factors and processes that affected the delivery, receipt, and outcome of the study to aid the interpretation and translation of the observed findings. We examined the following processes:
1) Reach: we collected anonymised data on those who were invited to take part but did not respond, to analyse participation biases during the screening stages. We collected the following anonymised data as potential predictors of response in a logistic regression: primary care surgery, QDiabetes risk score [33], BMI, age, gender, ethnicity, IMD 2015 decile, and HbA1c level.
2) Implementation processes: We administered a detailed process questionnaire (contact authors for access) at six-month and 12-month follow-ups assessing which aspects of the wristband, smartphone application, e-DPP, and text messaging that participants had found most or least helpful. Questions were a mixture of Likert-scale and open-ended ones. The intervention arm received several additional questions specifically about the text messaging aspect. We provide a descriptive summary of the responses and qualitative interpretation.
3) We conducted three focus groups with participants (see below).
4) We describe the technical and mechanical issues developed by the wristbands as reported by participants. We logged the number of additional wristbands given to participants as replacements and if any wristbands were lost, faulty, or damaged."

12a-i) Imputation techniques to deal with attrition / missing values

Imputation techniques to deal with attrition / missing values: Not all participants will use the intervention/comparator as intended and attrition is typically high in ehealth trials. Specify how participants who did not use the application or dropped out from the trial were treated in the statistical analysis (a complete case analysis is strongly discouraged, and simple imputation techniques such as LOCF may also be problematic [4]).

	1	2	3	4	5	
subitem not at all important	○	○	○	○	○	essential

Does your paper address subitem 12a-i? *

Copy and paste relevant sections from the manuscript (include quotes in quotation marks "like this" to indicate direct quotes from your manuscript), or elaborate on this item by providing additional information not in the ms, or briefly explain why the item is not applicable/relevant for your study

"The amount of missing data for individual items and entire measures were examined to determine the suitability of instruments and level of burden for a future full-scale trial."

"The following sensitivity analysis was conducted to determine if any violations of the assumption of data missing at random (MAR) affected the outcomes: for any demographic or clinical baseline variables that were predictors of outcome missingness, then those variables were included as covariates in the primary analysis model and post-treatment group difference estimates and associated confidence intervals were examined for differences."

"We conducted a second post hoc sensitivity analysis where weight and the secondary outcomes were re-analysed after excluding participants with missing PA outcome data."

12b) Methods for additional analyses, such as subgroup analyses and adjusted analyses

Does your paper address CONSORT subitem 12b? *

Copy and paste relevant sections from the manuscript (include quotes in quotation marks "like this" to indicate direct quotes from your manuscript), or elaborate on this item by providing additional information not in the ms, or briefly explain why the item is not applicable/relevant for your study

See 12a (above).

X26) REB/IRB Approval and Ethical Considerations [recommended as subheading under "Methods"] (not a CONSORT item)

X26-i) Comment on ethics committee approval

	1	2	3	4	5	
subitem not at all important	○	○	○	○	○	essential

Does your paper address subitem X26-i?

Copy and paste relevant sections from the manuscript (include quotes in quotation marks "like this" to indicate direct quotes from your manuscript), or elaborate on this item by providing additional information not in the ms, or briefly explain why the item is not applicable/relevant for your study

"The trial has been reviewed and given favourable opinion by the London City and East Research Ethics Committee (16/LO/1505) and approval has been granted by the Health Research Authority."

x26-ii) Outline informed consent procedures

Outline informed consent procedures e.g., if consent was obtained offline or online (how? Checkbox, etc.?), and what information was provided (see 4a-ii). See [6] for some items to be included in informed consent documents.

	1	2	3	4	5	
subitem not at all important	○	○	○	○	○	essential

Does your paper address subitem X26-ii?

Copy and paste relevant sections from the manuscript (include quotes in quotation marks "like this" to indicate direct quotes from your manuscript), or elaborate on this item by providing additional information not in the ms, or briefly explain why the item is not applicable/relevant for your study

The research team obtained participant consent offline. The consent form used checkboxes and included the following:

1. I confirm that I have read and understand the information sheet dated 23/09/2016 version 1.1 for the above study. I have had the opportunity to consider the information, ask questions and have had these answered satisfactorily.

2. I understand that my participation is voluntary and that I am free to withdraw at any time without giving any reason, without my medical care or legal rights being affected.

3. I agree to take part in the study

4. I agree for my medical notes to be checked by the research team for the duration of the study. I understand that the data collected during the study may be looked at by the study sponsor or the regulatory authorities. I give permission for these individuals to have access to my research data.

5. I agree for my data collected during the study to be shared with my general practitioner.

6. I agree for my data to be shared with the Buddi Ltd. company in order to set up the wearable technology and smartphone application. The company will not have access to personal identifiable information.

7. I agree to have blood samples taken as part of the study. Blood samples will be destroyed following analysis; no blood samples will be stored.

Please delete the following items if you do not wish to do them:

8. I agree to be contacted about ethically approved future research.

9. I agree to give verbal feedback to the research team about my participation in the study. I agree for feedback sessions, one to one or in a group, to be audiotaped and for direct quotes from the interviews to be used in reports. I understand that confidentiality will be maintained and it will not be possible to identify me from any publications.

10. I agree for my medical notes to be accessed by the research team/regulatory authorities for a period of up to 5 years after the end of the study.

11. I agree for my Hospital Episodes Statistics data, information on all hospital appointments for up to a 6 year period beginning 12 months before participating in the study, to be checked by the research team

12. I understand that information held by the NHS and records maintained by the NHS Information Centre and the NHS Central Register may be used to help contact me and provide information about my health status.

X26-iii) Safety and security procedures

Safety and security procedures, incl. privacy considerations, and any steps taken to reduce the likelihood or detection of harm (e.g., education and training, availability of a hotline)

	1	2	3	4	5	
subitem not at all important	○	○	○	○	○	essential

Does your paper address subitem X26-iii?

Copy and paste relevant sections from the manuscript (include quotes in quotation marks "like this" to indicate direct quotes from your manuscript), or elaborate on this item by providing additional information not in the ms, or briefly explain why the item is not applicable/relevant for your study

Your answer

RESULTS

13a) For each group, the numbers of participants who were randomly assigned, received intended treatment, and were analysed for the primary outcome

NPT: The number of care providers or centers performing the intervention in each group and the number of patients treated by each care provider in each center

Does your paper address CONSORT subitem 13a? *

Copy and paste relevant sections from the manuscript (include quotes in quotation marks "like this" to indicate direct quotes from your manuscript), or elaborate on this item by providing additional information not in the ms, or briefly explain why the item is not applicable/relevant for your study

See Figure 1. 200 participants were randomly assigned, 98 in the intervention arm and 102 in the control arm.

Intervention:
Primary intention-to-treat analyses for 12 months included 69 participants for weight (primary outcome) and 18 participants for physical activity (primary outcome).

Control:
Primary intention-to-treat analyses for 12 months included 87 participants for weight (primary outcome) and 31 participants for physical activity (primary outcome).

13b) For each group, losses and exclusions after randomisation, together with reasons

Does your paper address CONSORT subitem 13b? (NOTE: Preferably, this is shown in a CONSORT flow diagram) *

Copy and paste relevant sections from the manuscript (include quotes in quotation marks "like this" to indicate direct quotes from your manuscript), or elaborate on this item by providing additional information not in the ms, or briefly explain why the item is not applicable/relevant for your study

"Table 2 summarises the reasons for withdrawals from the intervention. Reasons for withdrawal did not differ between the arms at six- (□2(5)=6.29, P=0.28) or 12-month follow-up (□2(6)=7.68, P=0.26).

We further tested if the distribution of withdrawals, non-contactable participants, and intervention completers differed between arms and found a significant difference (□2(2)=9.69, P=0.008), with the control arm having a higher proportion of completers (80.4% v. 65.3%) and lower rate of non-contactable participants (2.0% v. 12.2%) and withdrawals (17.6% v. 22.4%)."

"For each of the six self-report measures (AUDIT, smoking status, PHQ-9, SEE, URICA, and IPAQ), we examined rates of missing item-level data. At baseline and six-month follow-up, there were low levels of missing item-level data (≤6.1% of responses). And at 12 months, there were very low levels of missing data (≤1.8% of responses)."

13b-i) Attrition diagram

Strongly recommended: An attrition diagram (e.g., proportion of participants still logging in or using the intervention/comparator in each group plotted over time, similar to a survival curve) or other figures or tables demonstrating usage/dose/engagement.

	1	2	3	4	5	
subitem not at all important	○	○	○	○	○	essential

Does your paper address subitem 13b-i?

Copy and paste relevant sections from the manuscript or cite the figure number if applicable (include quotes in quotation marks "like this" to indicate direct quotes from your manuscript), or elaborate on this item by providing additional information not in the ms, or briefly explain why the item is not applicable/relevant for your study

Paper does not include an attrition diagram.

14a) Dates defining the periods of recruitment and follow-up

Does your paper address CONSORT subitem 14a? *

Copy and paste relevant sections from the manuscript (include quotes in quotation marks "like this" to indicate direct quotes from your manuscript), or elaborate on this item by providing additional information not in the ms, or briefly explain why the item is not applicable/relevant for your study

"First invitation was sent in October 2016. Second invitation was sent in January 2017 where surgery target of 20 was reached."

The first participant was seen in December 2016 for their baseline appointment. The last participant was seen for their 12 month follow up appointment in June 2018.

"Three focus groups with participants who: i) received the intervention, ii) were in the control group, and iii) dropped out of the intervention, were conducted in July 2018."

14a-i) Indicate if critical “secular events” fell into the study period

Indicate if critical “secular events” fell into the study period, e.g., significant changes in Internet resources available or “changes in computer hardware or Internet delivery resources”

	1	2	3	4	5	
subitem not at all important	○	○	○	○	○	essential

Does your paper address subitem 14a-i?

Copy and paste relevant sections from the manuscript (include quotes in quotation marks "like this" to indicate direct quotes from your manuscript), or elaborate on this item by providing additional information not in the ms, or briefly explain why the item is not applicable/relevant for your study

No critical "secular events" fell into the study.

14b) Why the trial ended or was stopped (early)

Does your paper address CONSORT subitem 14b? *

Copy and paste relevant sections from the manuscript (include quotes in quotation marks "like this" to indicate direct quotes from your manuscript), or elaborate on this item by providing additional information not in the ms, or briefly explain why the item is not applicable/relevant for your study

The trial ended when the last participant was seen for their 12 month follow up appointment.

15) A table showing baseline demographic and clinical characteristics for each group

NPT: When applicable, a description of care providers (case volume, qualification, expertise, etc.) and centers (volume) in each group

Does your paper address CONSORT subitem 15? *

Copy and paste relevant sections from the manuscript (include quotes in quotation marks "like this" to indicate direct quotes from your manuscript), or elaborate on this item by providing additional information not in the ms, or briefly explain why the item is not applicable/relevant for your study

Please see Table 1.

15-i) Report demographics associated with digital divide issues

In ehealth trials it is particularly important to report demographics associated with digital divide issues, such as age, education, gender, social-economic status, computer/Internet/ehealth literacy of the participants, if known.

	1	2	3	4	5	
subitem not at all important	○	○	○	○	○	essential

Does your paper address subitem 15-i? *

Copy and paste relevant sections from the manuscript (include quotes in quotation marks "like this" to indicate direct quotes from your manuscript), or elaborate on this item by providing additional information not in the ms, or briefly explain why the item is not applicable/relevant for your study

Please see Table 1. Age, gender, education and social-economic status are presented.

16) For each group, number of participants (denominator) included in each analysis and whether the analysis was by original assigned groups

16-i) Report multiple “denominators” and provide definitions

Report multiple “denominators” and provide definitions: Report N’s (and effect sizes) “across a range of study participation [and use] thresholds” [1], e.g., N exposed, N consented, N used more than x times, N used more than y weeks, N participants “used” the intervention/comparator at specific pre-defined time points of interest (in absolute and relative numbers per group). Always clearly define “use” of the intervention.

	1	2	3	4	5	
subitem not at all important	○	○	○	○	○	essential

Does your paper address subitem 16-i? *

Copy and paste relevant sections from the manuscript (include quotes in quotation marks "like this" to indicate direct quotes from your manuscript), or elaborate on this item by providing additional information not in the ms, or briefly explain why the item is not applicable/relevant for your study

Each table includes the number of participants included in the analyses.

16-ii) Primary analysis should be intent-to-treat

Primary analysis should be intent-to-treat, secondary analyses could include comparing only “users”, with the appropriate caveats that this is no longer a randomized sample (see 18-i).

	1	2	3	4	5	
subitem not at all important	○	○	○	○	○	essential

Does your paper address subitem 16-ii?

Copy and paste relevant sections from the manuscript (include quotes in quotation marks "like this" to indicate direct quotes from your manuscript), or elaborate on this item by providing additional information not in the ms, or briefly explain why the item is not applicable/relevant for your study

Primary intention-to-treat analyses is displayed in Figure 1.

17a) For each primary and secondary outcome, results for each group, and the estimated effect size and its precision (such as 95% confidence interval)

Does your paper address CONSORT subitem 17a? *

Copy and paste relevant sections from the manuscript (include quotes in quotation marks "like this" to indicate direct quotes from your manuscript), or elaborate on this item by providing additional information not in the ms, or briefly explain why the item is not applicable/relevant for your study

The paper includes this.

17a-i) Presentation of process outcomes such as metrics of use and intensity of use

In addition to primary/secondary (clinical) outcomes, the presentation of process outcomes such as metrics of use and intensity of use (dose, exposure) and their operational definitions is critical. This does not only refer to metrics of attrition (13-b) (often a binary variable), but also to more continuous exposure metrics such as “average session length”. These must be accompanied by a technical description how a metric like a “session” is defined (e.g., timeout after idle time) [1] (report under item 6a).

	1	2	3	4	5	
subitem not at all important	○	○	○	○	○	essential

Does your paper address subitem 17a-i?

Copy and paste relevant sections from the manuscript (include quotes in quotation marks "like this" to indicate direct quotes from your manuscript), or elaborate on this item by providing additional information not in the ms, or briefly explain why the item is not applicable/relevant for your study

The paper does not include the presentation of process outcomes.

17b) For binary outcomes, presentation of both absolute and relative effect sizes is recommended

Does your paper address CONSORT subitem 17b? *

Copy and paste relevant sections from the manuscript (include quotes in quotation marks "like this" to indicate direct quotes from your manuscript), or elaborate on this item by providing additional information not in the ms, or briefly explain why the item is not applicable/relevant for your study

The paper does not include this.

18) Results of any other analyses performed, including subgroup analyses and adjusted analyses, distinguishing pre-specified from exploratory

Does your paper address CONSORT subitem 18? *

Copy and paste relevant sections from the manuscript (include quotes in quotation marks "like this" to indicate direct quotes from your manuscript), or elaborate on this item by providing additional information not in the ms, or briefly explain why the item is not applicable/relevant for your study

These have been included in the paper.

18-i) Subgroup analysis of comparing only users

A subgroup analysis of comparing only users is not uncommon in ehealth trials, but if done, it must be stressed that this is a self-selected sample and no longer an unbiased sample from a randomized trial (see 16-iii).

	1	2	3	4	5	
subitem not at all important	○	○	○	○	○	essential

Does your paper address subitem 18-i?

Copy and paste relevant sections from the manuscript (include quotes in quotation marks "like this" to indicate direct quotes from your manuscript), or elaborate on this item by providing additional information not in the ms, or briefly explain why the item is not applicable/relevant for your study

Your answer

19) All important harms or unintended effects in each group

(for specific guidance see CONSORT for harms)

Does your paper address CONSORT subitem 19? *

Copy and paste relevant sections from the manuscript (include quotes in quotation marks "like this" to indicate direct quotes from your manuscript), or elaborate on this item by providing additional information not in the ms, or briefly explain why the item is not applicable/relevant for your study

There are no important harms or unintended effects to report.

19-i) Include privacy breaches, technical problems

Include privacy breaches, technical problems. This does not only include physical “harm” to participants, but also incidents such as perceived or real privacy breaches [1], technical problems, and other unexpected/unintended incidents. “Unintended effects” also includes unintended positive effects [2].

	1	2	3	4	5	
subitem not at all important	○	○	○	○	○	essential

Does your paper address subitem 19-i?

Copy and paste relevant sections from the manuscript (include quotes in quotation marks "like this" to indicate direct quotes from your manuscript), or elaborate on this item by providing additional information not in the ms, or briefly explain why the item is not applicable/relevant for your study

"Fifty-seven participants (30 in the intervention arm) reported their wristbands developing some type of technical or mechanical fault. The primary faults reported were the wristband not charging or being unresponsive (i.e., not vibrating/flashing when buttons were pushed or connecting to the smartphone application). And, 24 participants (10 in the intervention arm) reported damaging their wristband and requiring a replacement. The wristbands were mainly damaged because the buttons fell off, the protective casing was broken, or water ingress damaged the inner circuits."

19-ii) Include qualitative feedback from participants or observations from staff/researchers

Include qualitative feedback from participants or observations from staff/researchers, if available, on strengths and shortcomings of the application, especially if they point to unintended/unexpected effects or uses. This includes (if available) reasons for why people did or did not use the application as intended by the developers.

	1	2	3	4	5	
subitem not at all important	○	○	○	○	○	essential

Does your paper address subitem 19-ii?

Copy and paste relevant sections from the manuscript (include quotes in quotation marks "like this" to indicate direct quotes from your manuscript), or elaborate on this item by providing additional information not in the ms, or briefly explain why the item is not applicable/relevant for your study

"The focus groups included 14 participants (n=8 males, n=6 female) aged between 49 and 65 years. Four of the invited participants did not attend. Four main themes emerged: awareness, quality of technology, self-efficacy and behaviour change. There are facets of the participants' views that overlap across these themes."

DISCUSSION

22) Interpretation consistent with results, balancing benefits and harms, and considering other relevant evidence

NPT: In addition, take into account the choice of the comparator, lack of or partial blinding, and unequal expertise of care providers or centers in each group

22-i) Restate study questions and summarize the answers suggested by the data, starting with primary outcomes and process outcomes (use)

Restate study questions and summarize the answers suggested by the data, starting with primary outcomes and process outcomes (use).

	1	2	3	4	5	
subitem not at all important	○	○	○	○	○	essential

Does your paper address subitem 22-i? *

Copy and paste relevant sections from the manuscript (include quotes in quotation marks "like this" to indicate direct quotes from your manuscript), or elaborate on this item by providing additional information not in the ms, or briefly explain why the item is not applicable/relevant for your study

"We have conducted a pilot RCT which tested the effectiveness of an intervention integrating a smartphone application that delivered an online educational DPP, with text messages incorporating motivational interviewing techniques and biofeedback via a wearable device over a period of 12 months in reducing the risk of type 2 diabetes in pre-diabetes patients.

We successfully recruited to our target sample size in 36 weeks with a recruitment rate of 5-6 patients per week per full-time research worker. For a full-scale RCT testing the intervention with an expected effect size of 0.1 and 90% power, we estimate recruitment will take about 3.5 years (Table 4).

We also successfully recruited a generalisable sample of participants representative of age, gender, ethnicity, and socioeconomic deprivation. However, we did find that patients whose surgeries were in more deprived areas were less likely to respond to the invitation letters.

We piloted our statistical plan that we would anticipate using for a full-scale RCT and this appeared to be valid, although hypothesis-testing was only a secondary aim. We observed only a few minor differences between the arms, with the only significant treatment difference that those in the intervention had lower PA at six months. However, intervention arm participants were also less likely to complete the intervention and more likely to be non-contactable or have withdrawn at follow-up."

22-ii) Highlight unanswered new questions, suggest future research

Highlight unanswered new questions, suggest future research.

	1	2	3	4	5	
subitem not at all important	○	○	○	○	○	essential

Does your paper address subitem 22-ii?

Copy and paste relevant sections from the manuscript (include quotes in quotation marks "like this" to indicate direct quotes from your manuscript), or elaborate on this item by providing additional information not in the ms, or briefly explain why the item is not applicable/relevant for your study

"We have piloted a model e-health intervention to support diabetes prevention programmes in preparation for a full-scale RCT."

20) Trial limitations, addressing sources of potential bias, imprecision, and, if relevant, multiplicity of analyses

20-i) Typical limitations in ehealth trials

Typical limitations in ehealth trials: Participants in ehealth trials are rarely blinded. Ehealth trials often look at a multiplicity of outcomes, increasing risk for a Type I error. Discuss biases due to non-use of the intervention/usability issues, biases through informed consent procedures, unexpected events.

	1	2	3	4	5	
subitem not at all important	○	○	○	○	○	essential

Does your paper address subitem 20-i? *

Copy and paste relevant sections from the manuscript (include quotes in quotation marks "like this" to indicate direct quotes from your manuscript), or elaborate on this item by providing additional information not in the ms, or briefly explain why the item is not applicable/relevant for your study

"Despite a prior 'road-test' of the technology by the commercial provider in a handful of volunteers, there were mechanical and technical failures that may have resulted in participants randomised to the intervention arm in receiving less of the 'dose' and withdrawing because of these issues. For example, we observed a sharp decrease in the mean step counts for participants at six- and 12-month follow-up compared to baseline, suggesting that the wristband may not have accurately recorded activity over time or perhaps that participants were unaware of ongoing technical issues.

Participant adherence to the intervention was not comprehensively documented. The Buddi device recorded participants' daily duration of using the wristband (i.e., how many hours of each day the device captured activity). However, we were not able to capture participants' usage of the application, particularly, if they accessed the DPP education materials (and for how long) or if they read the SMS messages sent to them."

"We assessed the degree that the wristband-derived step counts (i.e., objectively measured physical activity) were associated with a validated self-reported measure of physical activity levels. We observed that the two measures only corresponded to a limited extent in the control arm, with step counts being weakly, positively correlated with total self-reported activity at two time points. The self-reported scores were not associated with step counts in the intervention arm and there are several possible reasons for this discrepancy, one being that these participants became disengaged from the intervention or experienced more issues with the smartphone application due to the higher intensity, thereby potentially wearing the wristband less frequently."

21) Generalisability (external validity, applicability) of the trial findings

NPT: External validity of the trial findings according to the intervention, comparators, patients, and care providers or centers involved in the trial

21-i) Generalizability to other populations

Generalizability to other populations: In particular, discuss generalizability to a general Internet population, outside of a RCT setting, and general patient population, including applicability of the study results for other organizations

	1	2	3	4	5	
subitem not at all important	○	○	○	○	○	essential

Does your paper address subitem 21-i?

Copy and paste relevant sections from the manuscript (include quotes in quotation marks "like this" to indicate direct quotes from your manuscript), or elaborate on this item by providing additional information not in the ms, or briefly explain why the item is not applicable/relevant for your study

"We also successfully recruited a generalisable sample of participants representative of age, gender, ethnicity, and socioeconomic deprivation."

21-ii) Discuss if there were elements in the RCT that would be different in a routine application setting

Discuss if there were elements in the RCT that would be different in a routine application setting (e.g., prompts/reminders, more **human inv**olvement, training sessions or other co-interventions) and what impact the omission of these elements could have on use, adoption, or outcomes if the intervention is applied outside of a RCT setting.

	1	2	3	4	5	
subitem not at all important	○	○	○	○	○	essential

Does your paper address subitem 21-ii?

Copy and paste relevant sections from the manuscript (include quotes in quotation marks "like this" to indicate direct quotes from your manuscript), or elaborate on this item by providing additional information not in the ms, or briefly explain why the item is not applicable/relevant for your study

Your answer

OTHER INFORMATION

23) Registration number and name of trial registry

Does your paper address CONSORT subitem 23? *

Copy and paste relevant sections from the manuscript (include quotes in quotation marks "like this" to indicate direct quotes from your manuscript), or elaborate on this item by providing additional information not in the ms, or briefly explain why the item is not applicable/relevant for your study

"Trial Registration: ClinicalTrials.gov NCT02919397."

24) Where the full trial protocol can be accessed, if available

Does your paper address CONSORT subitem 24? *

Cite a Multimedia Appendix, other reference, or copy and paste relevant sections from the manuscript (include quotes in quotation marks "like this" to indicate direct quotes from your manuscript), or elaborate on this item by providing additional information not in the ms, or briefly explain why the item is not applicable/relevant for your study

"The full trial protocol can be accessed from https://www.kcl.ac.uk/ioppn/depts/pm/people/acaprof/diabetes-stopwatch-protocol.pdf."

25) Sources of funding and other support (such as supply of drugs), role of funders

Does your paper address CONSORT subitem 25? *

Copy and paste relevant sections from the manuscript (include quotes in quotation marks "like this" to indicate direct quotes from your manuscript), or elaborate on this item by providing additional information not in the ms, or briefly explain why the item is not applicable/relevant for your study

"Funding for this study was provided by Innovate UK (ID: 21370). The funder had no role in the design of the study, or the collection, analysis, and interpretation of data, and no part in writing the manuscript. Buddi Ltd. provided the wristband and app."

X27) Conflicts of Interest (not a CONSORT item)

X27-i) State the relation of the study team towards the system being evaluated

In addition to the usual declaration of interests (financial or otherwise), also state the relation of the study team towards the system being evaluated, i.e., state if the authors/evaluators are distinct from or identical with the developers/sponsors of the intervention.

	1	2	3	4	5	
subitem not at all important	○	○	○	○	○	essential

Does your paper address subitem X27-i?

Copy and paste relevant sections from the manuscript (include quotes in quotation marks "like this" to indicate direct quotes from your manuscript), or elaborate on this item by providing additional information not in the ms, or briefly explain why the item is not applicable/relevant for your study

"KI has received honorarium for educational lectures for Janssen, Sanofi, Eli Lilly and Novo Nordisk. The other authors have none to declare."

About the CONSORT EHEALTH checklist

As a result of using this checklist, did you make changes in your manuscript? *

- ○ yes, major changes
- ◉ yes, minor changes
- ○ no

What were the most important changes you made as a result of using this checklist?

Your answer

How much time did you spend on going through the checklist INCLUDING making changes in your manuscript *

4 hours.

As a result of using this checklist, do you think your manuscript has improved? *

- (selected) yes
- no
- Other:

Would you like to become involved in the CONSORT EHEALTH group?

This would involve for example becoming involved in participating in a workshop and writing an "Explanation and Elaboration" document

- yes
- (selected) no
- Other:

Any other comments or questions on CONSORT EHEALTH

Your answer

STOP - Save this form as PDF before you click submit

To generate a record that you filled in this form, we recommend to generate a PDF of this page (on a Mac, simply select "print" and then select "print as PDF") before you submit it.

When you submit your (revised) paper to JMIR, please upload the PDF as supplementary file.

Don't worry if some text in the textboxes is cut off, as we still have the complete information in our database. Thank you!

Final step: Click submit !

Click submit so we have your answers in our database!

SUBMIT

Never submit passwords through Google Forms.

This content is neither created nor endorsed by Google. Report Abuse - Terms of Service

Google Forms
